# Exploring Gene Expression Signatures for Predicting Disease Free Survival after Resection of Colorectal Cancer Liver Metastases

**DOI:** 10.1371/journal.pone.0049442

**Published:** 2012-11-21

**Authors:** Nikol Snoeren, Sander R. van Hooff, Rene Adam, Richard van Hillegersberg, Emile E. Voest, Catherine Guettier, Paul J. van Diest, Maarten W. Nijkamp, Mariel O. Brok, Dik van Leenen, Marian J. A. Groot Koerkamp, Frank C. P. Holstege, Inne H. M. Borel Rinkes

**Affiliations:** 1 Department of Surgery, University Medical Center Utrecht, Heidelberglaan 100, Utrecht, The Netherlands; 2 Department of Molecular Cancer Research, University Medical Center Utrecht, Heidelberglaan 100, Utrecht, The Netherlands; 3 Department of Surgery, Centre Hépato-Biliaire, AP-HP Hôpital Paul Brousse, 12 Avenue Paul Vaillant Couturier, Villejuif, France; 4 Department of Medical Oncology, University Medical Center Utrecht, Heidelberglaan 100, Utrecht, The Netherlands; 5 Department of Pathology, Centre Hépato-Biliaire, AP-HP Hôpital Paul Brousse, 12 Avenue Paul Vaillant Couturier, Villejuif, France; 6 Department of Pathology, University Medical Center Utrecht, Heidelberglaan 100, Utrecht, The Netherlands; Baylor University Medical Center, United States of America

## Abstract

**Background and Objectives:**

This study was designed to identify and validate gene signatures that can predict disease free survival (DFS) in patients undergoing a radical resection for their colorectal liver metastases (CRLM).

**Methods:**

Tumor gene expression profiles were collected from 119 patients undergoing surgery for their CRLM in the Paul Brousse Hospital (France) and the University Medical Center Utrecht (The Netherlands). Patients were divided into high and low risk groups. A randomly selected training set was used to find predictive gene signatures. The ability of these gene signatures to predict DFS was tested in an independent validation set comprising the remaining patients. Furthermore, 5 known clinical risk scores were tested in our complete patient cohort.

**Result:**

No gene signature was found that significantly predicted DFS in the validation set. In contrast, three out of five clinical risk scores were able to predict DFS in our patient cohort.

**Conclusions:**

No gene signature was found that could predict DFS in patients undergoing CRLM resection. Three out of five clinical risk scores were able to predict DFS in our patient cohort. These results emphasize the need for validating risk scores in independent patient groups and suggest improved designs for future studies.

## Introduction

Colorectal cancer is the third most common cancer in men and the second in women worldwide, accounting for approximately 608.000 deaths worldwide [1]. The liver is the most common and often only site of metastatic disease. The development of liver metastases in about 50% of patients is the major determinant of survival in patients with colorectal cancer. Surgical resection is the best treatment option for patients with colorectal liver metastasis offering a median survival of over 40 months after resection compared to a median survival of 18 months when treated with chemotherapy and 6 to12 months if patients remain untreated [2]. Unfortunately, 60%–80% of patients will develop local or distant recurrences after R0 resection of colorectal liver metastasis [2]–[5]. Patients with recurrence are likely to benefit from adjuvant chemotherapy. However, 20–40% of the patients do not develop recurrence and will probably better be left untreated after liver resection. Since chemotherapy is associated with serious morbidity and mortality, the therapy-associated risk should therefore be justified by a significant improvement in survival of these patients.

Many research groups have attempted to define factors predicting disease free survival and overall survival (OS) after resection of liver metastasis [5], [6]. Recently, five published clinical risk scores, combining different clinical factors, were validated in an independent patient cohort demonstrating that two clinical risk scores were able to predict overall survival in an independent set of patients [7]. Prediction of (disease-free) survival might be improved by the use of gene expression which might capture tumor properties not reflected by clinicopathological variables.

Genome wide gene expression profiling has been used to predict disease outcome or response to therapy in many different tumor types [8], [9] It has also been shown that expression profiling can be used to identify colorectal tumors with different aggressiveness and metastatic potential [10]–[13]. No study, however, has been published in which gene expression was used to predict disease free survival after resection of colorectal liver metastasis. Identification of a gene signature able to identify recurrence-prone colorectal liver metastases at time of resection would open the way for selection of patients who are likely to benefit from aggressive therapy after resection, while withholding others unnecessary treatment.

**Table 1:** Patient- and tumor characteristics of high and low risk patients.^a^

Category	Subcategory	DFS ≤1 year	DFS >1 year	Total	*P* value^b^
Total number of patients		72	47	119	
Sex	Male	45 (62.5%)	32 (68.1%)	77 (64.7%)	0.534
	Female	27 (37.5%)	15 (31.9%)	42 (35.3%)	
Age (Mean, SD)		60.6 (12.8)	62.6 (9.0)	61.4 (11.43)	0.351
Location of primary tumor	Rectum	18 (25.0%)	12 (25.5%)	30 (25.2%)	0.948
	Colon	54 (75.0%)	35 (74.5%)	89 (74.8%)	
Differentiation primary tumor	Good	9 (12.5%)	7 (14.9%)	16 (13.4%)	0.633
	Moderate	52 (72.2%)	34 (72.3%)	86 (72.3%)	
	Poor	11 (15.3%)	6 (12.8%)	17 (14.3%)	
Nodal Status	N+	42 (58.3%)	24 (51.1%)	66 (55.5%)	0.530
	N−	23 (31.9%)	17 (36.2%)	40 (33.6%)	
	Missing	7 (9.7%)	6 (12.8%)	13 (10.9%)	
Interval primary tumor and LM	Metachronous (>2 months)	35 (48.6%)	26 (55.3%)	61 (51.3%)	0.475
	Synchronous (≤2 months)	37 (51.4%)	21 (44.7%)	58 (48.7%)	
Neoadjuvant chemotherapy	Yes	45 (62.5%)	19 (40.4%)	64 (53.8%)	0.019
	No	27 (37.5%)	28 (59.6%)	55 (46.2%)	
Type of resection	Minor (3 segments resected or less)	41 (56.9%)	35 (74.5%)	76 (63.9%)	0.054
	Major	31 (42.5%)	12 (26.1%)	43 (36.1%)	
R0/R1 Resection	R0	51 (70.8%)	37 (78.7%)	88 (73.9%)	0.472
	R1	19 (26.4%)	10 (21.3%)	29 (24.4%)	
	Missing	2 (2.8%)		2 (1.7%)	
Bloodtransfusion	No	50 (69.4%)	36 (76.6%)	86 (72.3%)	0.350
	Yes	21 (29.2%)	10 (21.3%)	31 (26.1%)	
	Missing	1 (1.4%)	1 (2.1%)	2 (1.7%)	
Distribution	Bilobar	31 (43.1%)	19 (40.4%)	50 (42%)	0.851
	Unilobar	41 (56.9%)	27 (57.4%)	68 (57%)	
	Missing		1 (2.1%)	1 (0.8%)	
Mean number of LM/Patient		2.88 (2.80)	2.13 (1.62)	2.58 (2.43)	0.112
Tumorsize biggest metastases (cm)		5.25 (3.35)	4.43 (2.83)	4.93 (3.17)	0.172
Tumor cell percentage (Mean, SD)		45.44 (24.43)	44.77 (22.55)	45.18 (23.62)	0.884
Necrosis percentage (Mean, SD)		18.13 (15.05)	21.17 (20.30)	19.33 (17.30)	0.349
Fibrosis percentage (Mean, SD)		18.13 (15.05)	22.11 (20.24)	19.66 (17.25)	0.227
Preoperative CEA (Mean, SD)		84.35 (117.3)	73.27 (183.8)	79.98 (146.36)	0.706
Postoperative CEA (Mean, SD)		18.78 (63.91)	3.07 (6.57)	12.57 (50.27)	0.141
Adjuvant chemotherapy	Yes	33 (45.8%)	35 (74.5%)	68 (57.1%)	0.003
	No	39 (54.2%)	12 (25.5%)	51 (42.9%)	

DFS, disease free survival; LM, lymph nodes; CEA, carcinoembryonic antigen.

a Percentages may not total 100 because of rounding.

b *P* values were calculated with the use of Mann-Whitney test for continuous variables and Fisher’s exact test for categorical variables.

## Results

### Patients and Tumor Samples

Hundred forty-eight patients met the in- and exclusion criteria expression. Profiles were successfully obtained for 119 patients. The baseline characteristics of the 119 included patients, shown in Table 1, did not differ significantly between the high versus low risk group, with the exception of administration of chemotherapy. High-risk patients received neoadjuvant chemotherapy more frequently and adjuvant chemotherapy less frequently than low-risk patients. Patient samples had a mean tumor cell percentage of 45% (95%CI 40.75–49.60), necrosis 19% (95%CI 16.19–22.47) and fibrosis 20% (95%CI 16.44–22.71). Mean follow up was 26.7 months. A comparison of the baseline characteristics of the 119 included and 29 excluded patients is shown in Table S1.

**Figure 1:**
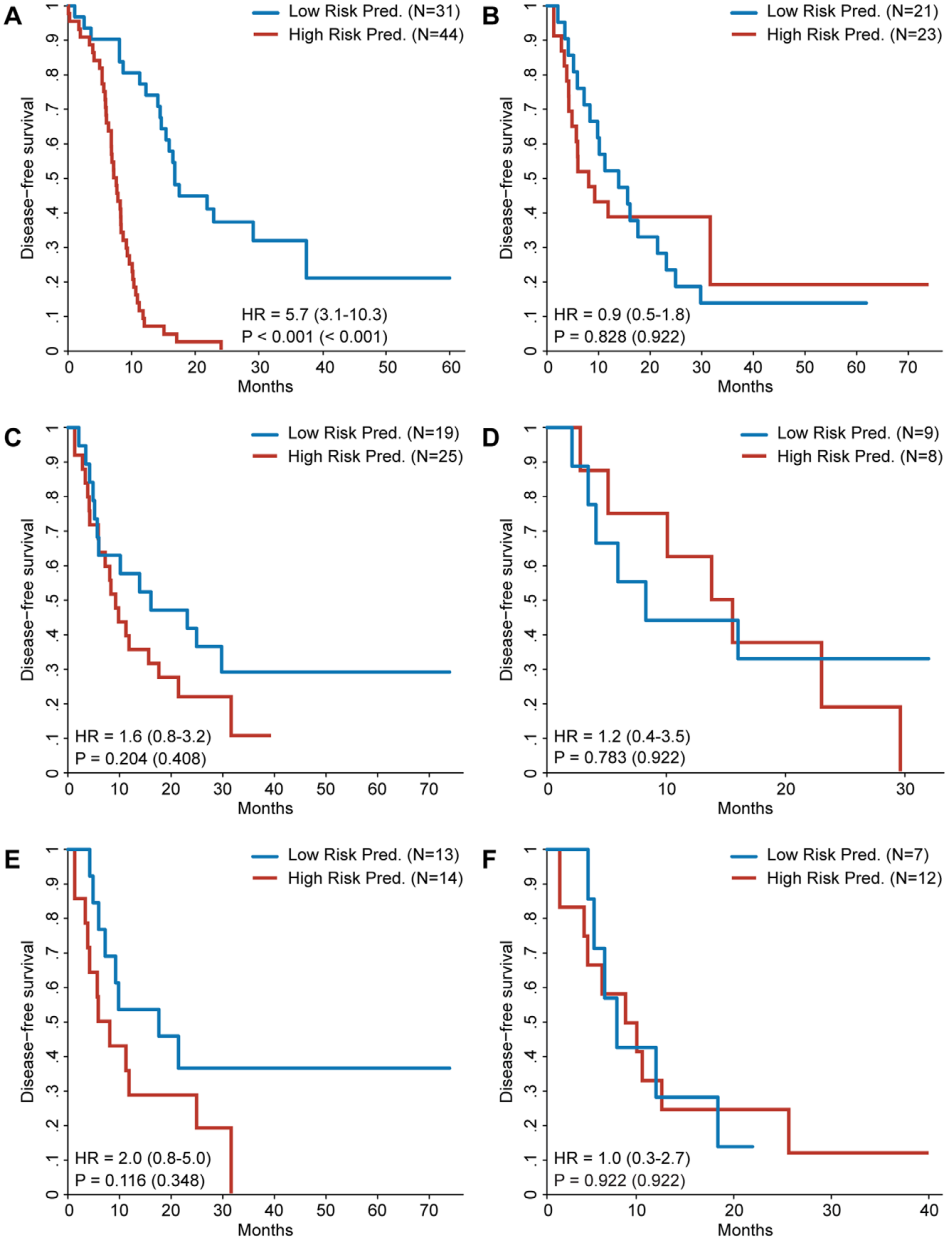
Kaplan–Meier survival analysis for the discovered gene signatures. Patients are divided into a high and a low risk prediction group based on the risk prediction of the different gene signatures. Gene signatures were discovered defining high risk as DFS ≤1 year and low risk as DFS >1 year unless mentioned otherwise. The hazard ratio of the gene signature prediction is shown with the 95% confidence interval between brackets. The p value of the log-rank test is shown as well, with the p value adjusted for multiple testing between brackets. **A:** Survival curves for patients in training set. Gene signature was discovered using the same training set. **B:** Survival curves for patients in the validation set. Gene signature was discovered using the full training set. **C:** Survival curves for patients in the validation set. Gene signature was discovered using the full training set defining high risk as DFS ≤6 months and low risk as DFS >2 years. **D:** Survival curves for UMC Utrecht patients in the validation set. Gene signature was discovered using the UMC Utrecht subset of the training set. **E:** Survival curves for Paul Brousse patients in the validation set. Gene signature was discovered using the Paul Brousse subset of the training set. **F:** Like E but including only Paul Brousse patients who received neoadjuvant chemotherapy (training and validation set).

**Figure 2:**
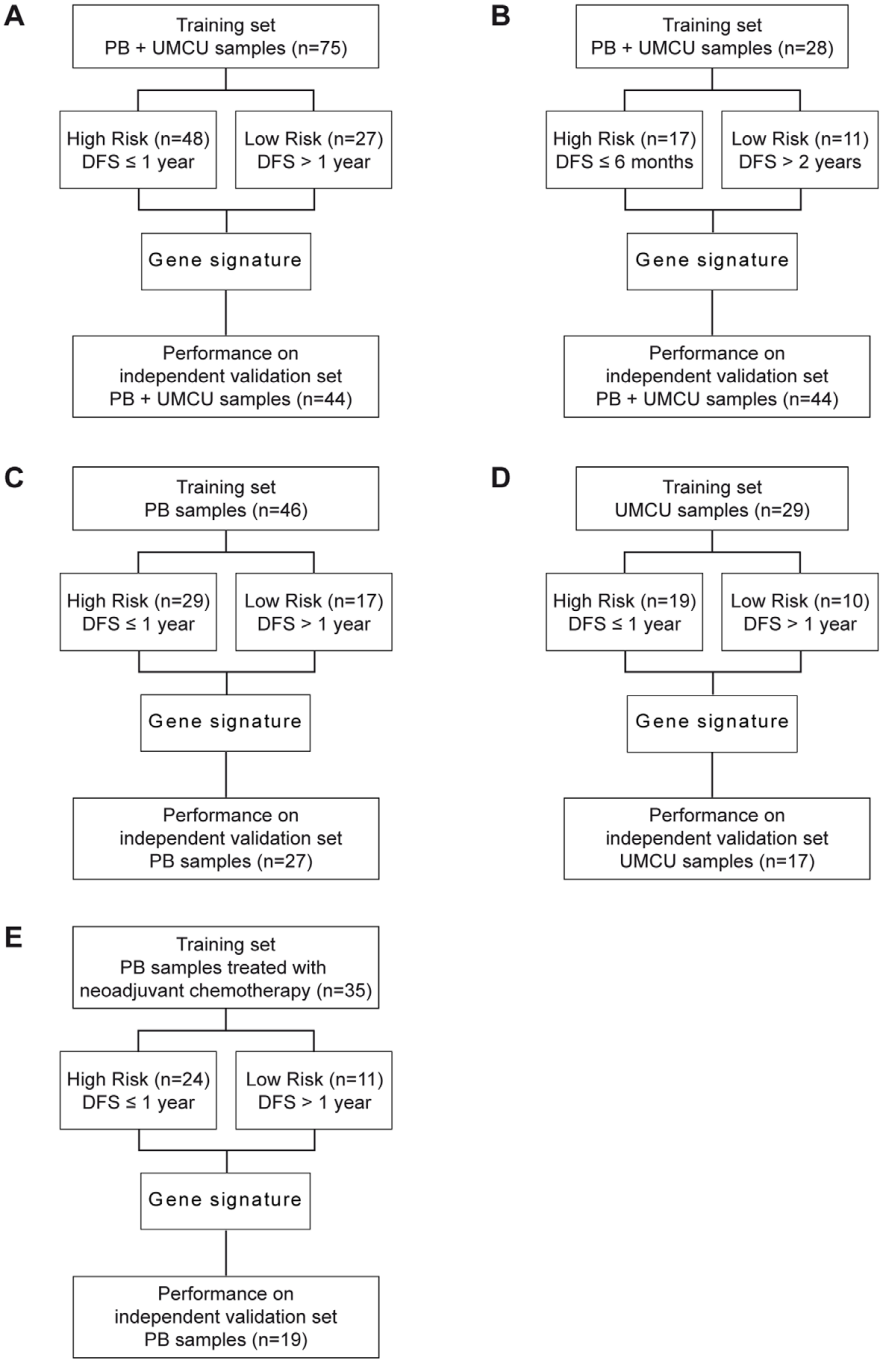
Flow charts showing the study design. A: Original set up of the study: supervised model dividing patients with DFS ≤1 year versus patients with DFS >1 year. The gene signature was discovered using the training set and subsequently tested on the independent validation set. **B:** Similar to A, using a supervised model dividing patients with DFS ≤6 months versus patients with DFS >2 years. **C:** Similar to A, including only patients treated in Paul Brousse. **D:** Similar to A, including only patients treated in UMC Utrecht. **E:** Similar to A, including only patients treated in Paul Brousse treated with neoadjuvant chemotherapy.

### Gene Expression Signature

Using the training set of 75 patients from both centers, a gene signature was discovered consisting of 20 genes (Table S2). This was the most predictive gene signature as measured within the training set, able to predict disease free survival with high statistical significance (Figure 1A). When used to predict risk for the patients in the independent validation set of 44 patients, however, this gene signature was unable to significantly predict DFS (Figure 1B). This points to overfitting on the training set patients, a fact underscored by the area under the curve (AUC) of 0.508 (95%CI 0.482–0.534) achieved during the signature discovery (see Methods). The power of the log-rank test used is shown in Figure S1. An analysis to find functional enrichment for the 20 genes in the signature failed to find any significant enrichment. Having failed to find a predictive gene signature, we examined whether a stricter definition of the high and low risk groups would result in a better gene signature by dividing the training set into a high risk group of patients with a DFS less than 6 months and a low risk group with a DFS of at least 2 years (Figure 2B). Although the validation results of this gene signature seemed to show a positive trend it also failed to reach significance (Figure 1C).

**Figure 3:**
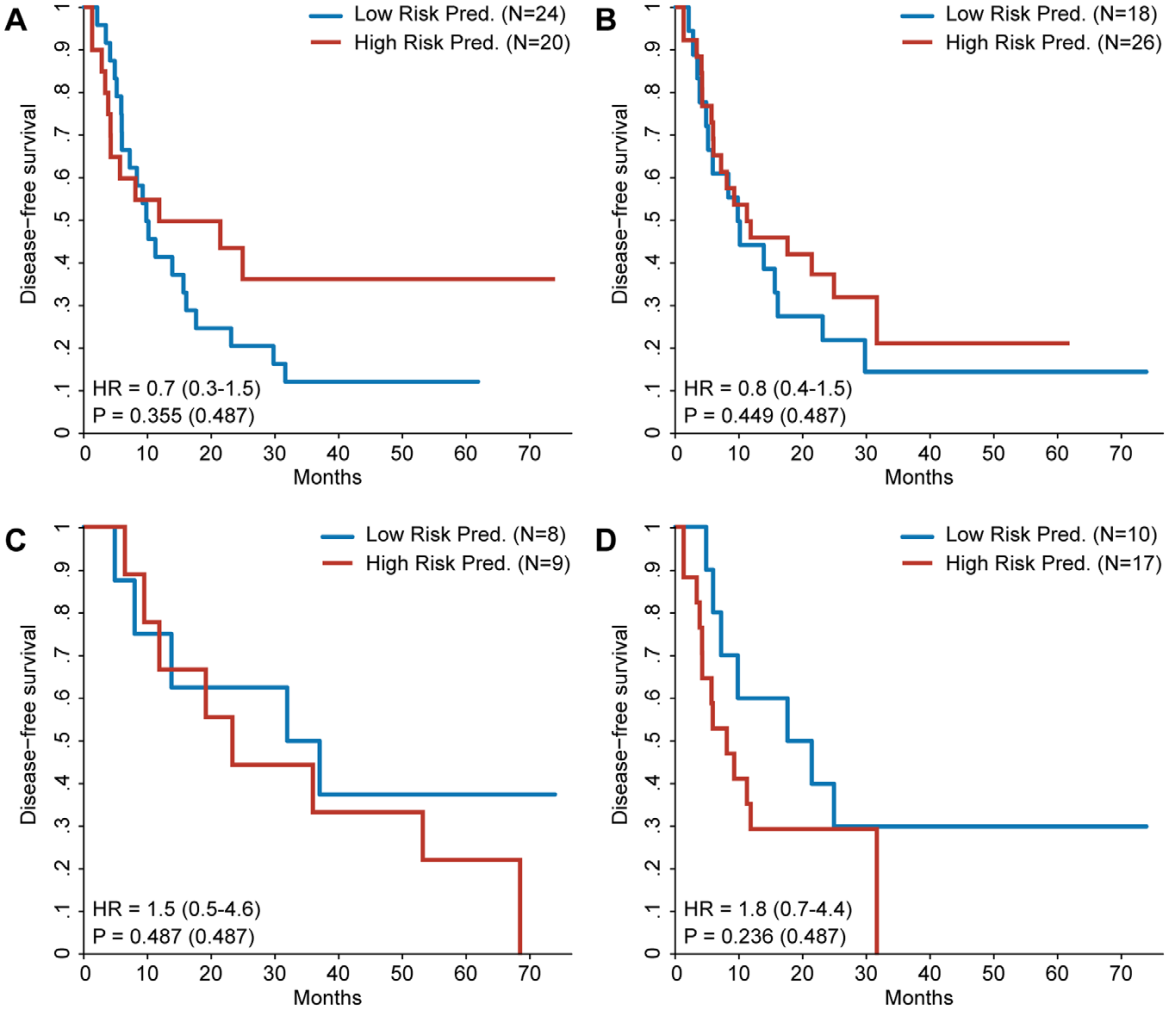
Kaplan–Meier survival analysis for gene signatures based on training sets without neoadjuvant treatment bias. Patients are divided into a high and a low risk prediction group based on the risk prediction of the different gene signatures. Gene signatures were discovered defining high risk as DFS ≤1 year and low risk as DFS >1 year unless mentioned otherwise. In all training sets the ratio of patients treated with neoadjuvant chemotherapy to untreated patients in high and low risk group was kept as equal as possible to preclude any treatment bias. The hazard ratio of the gene signature prediction is shown with the 95% confidence interval between brackets. The p value of the log-rank test is shown as well, with the p value adjusted for multiple testing between brackets. **A:** Survival curves for patients in the validation set. Gene signature was discovered using the full training set controlled for the neoadjuvant treatment bias. **B:** Survival curves for patients in the validation set. Gene signature was discovered using the full training set defining high risk as DFS ≤6 months and low risk as DFS >2 years and controlling for the neoadjuvant treatment bias. **C:** Survival curves for UMC Utrecht patients in the validation set. Gene signature was discovered using the UMC Utrecht subset of the training set controlled for the neoadjuvant treatment bias. **D:** Survival curves for Paul Brousse patients in the validation set. Gene signature was discovered using the Paul Brousse subset of the training set controlled for the neoadjuvant treatment bias.

**Figure 4:**
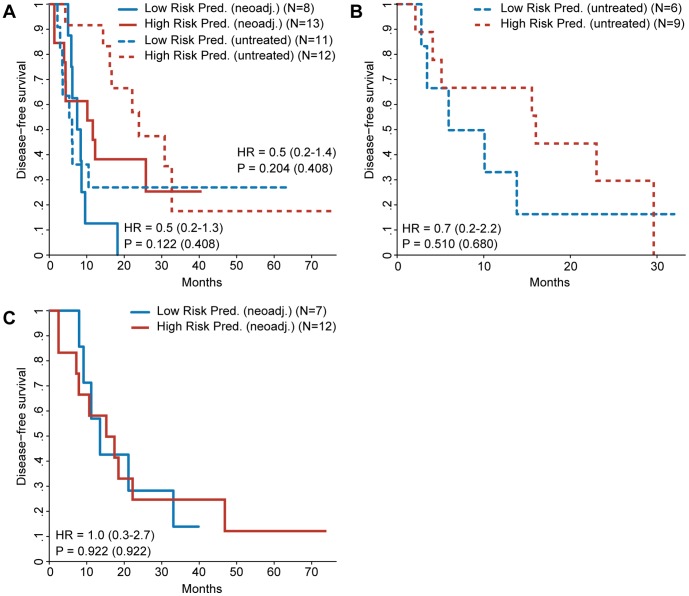
Kaplan–Meier survival analysis for gene signatures based on training sets stratified according to neoadjuvant treatment. Patients are divided into a high and a low risk prediction group based on the risk prediction of the different gene signatures. Gene signatures were discovered defining high risk as DFS ≤1 year and low risk as DFS >1 year unless mentioned otherwise. Both training and validation sets were separated into neoadjuvant treated and untreated patients. Results are only shown where the training sets contained enough high and low risk patients to make signature discovery possible. The hazard ratio of the gene signature prediction is shown with the 95% confidence interval between brackets. The p value of the log-rank test is shown as well, with the p value adjusted for multiple testing between brackets. **A:** Survival curves for patients in the validation set. Gene signatures were discovered using the full training set stratified by neoadjuvant treatment. **B:** Survival curves for untreated UMC Utrecht patients in the validation set. Gene signature was discovered using untreated UMC Utrecht patients in the training set. **C:** Survival curves for neoadjuvant treated Paul Brousse patients in the validation set. Gene signature was discovered using neoadjuvant treated Paul Brousse patients of the training set.

**Table 2:** Univariate and multivariate Cox regression analysis for risk factors associated with DFS (months) in Paul Brousse validation set.

Variable	Univariate^a^			Multivariate^b^		
	P value^c^	HR	95% CI	P value^c^	HR	95% CI
Neoadjuvantchemotherapy	0.046	5.32	1.02–9.96	0.083	3.87	0.84–17.79
Stage primarytumor^d^	0.003	11.09	1.43–85.70	0.028	9.90	1.27–77.02
Gene signatureprediction	0.12	2.03	0.83–5.01	0.69	1.25	0.42–3.72

DFS, disease free survival; HR, hazard ratio; CI, confidence interval.

a Only showing factors with p≤0.05 as well as Gene signature prediction.

b Multivariate model includes factors with p≤0.05 in univariate analysis (Neoadjuvant chemotherapy and Stage primary tumor) as well as Gene signature prediction.

c P values were calculated with the use of log-rank test.

d TNM stages 1 and 2 versus 3 and 4.

Some of the clinicopathological factors differed significantly between the patients from the Paul Brousse Hospital and the University Medical Center in Utrecht (Table S3). To explore the possibility that the previous failure to find a predictive gene signature might have been caused by these differences, the gene signature discovery was repeated for the UMC Utrecht samples and the Paul Brousse samples separately. The gene signature derived from the UMC Utrecht data alone did not hold any predictive power when validated (Figure 1D). Validation of the Paul Brousse gene signature, however, did show a positive trend (Figure 1E). The result of a multivariate Cox regression, however, suggests that the gene signature is not an independent predictive factor (Table 2). Stage of the primary tumor and the administration of neoadjuvant chemotherapy seemed sufficient to predict DFS within the validation set. It is possible that neoadjuvant chemotherapy, which is administered before the sample collection, had an effect on the gene expression pattern and was therefore an interfering factor in the experimental setup. This is confirmed by the absence of predictive power when the signature discovery was performed exclusively on Paul Brousse patients who did receive neoadjuvant chemotherapy (Figure 1F). Additionally, an analysis of the genes differentially expressed between patients treated with neoadjuvant chemotherapy and untreated patients revealed 875 genes that were significantly up- or downregulated (Table S4) suggesting that neoadjuvant chemotherapy induces a sizeable change in the measured gene expression. To investigate whether the absence of a predictive signature was caused by the neoadjuvant treatment bias in the high risk group the signature discovery was repeated using training sets were this bias was removed (Figure 3) as well as analyzing the neoadjuvant treated and untreated patients separately (Figure 4). The results strongly suggest that the absence of a predictive signature is independent of the effects of neoadjuvant treatment, adding the caveat that in some of these comparisons the sample size is low. Table S5 shows the predictive performance of all the gene signatures described above when used to predict DFS redefined as a dichotomous variable.

**Table 3:** Univariate Cox regression analysis for possible risk factors associated with DFS (months).

Category	Subcategory	Mean DFS (95%CI)	HR	95%CI HR	P value^a^
Sex	Male	18.61 (13.49–23.72)	1.082	0.707–1.654	0.718
	Female	19.12 (12.55–25.71)			
Age	≤65	17.87 (12.64–23.09)	0.986	0.556–1.264	0.399
	>65	16.67 (12.64–20.70)			
Location of primary tumor	Rectum	21.63 (12.16–31.10)	0.989	0.623–1.570	0.963
	Colon	17.21 (13.19–21.23)			
Differentiation primary tumor	Good	15.82 (5.94–25.70)	1.219	0.672–2.210	0.514
	Moderate	19.54 (14.22–24.86)	0.899	0.680–1.189	0.456
	Poor	12.73 (7.34–18.12)			
Stage primary tumor	1	35.23 (18.21–52.24)	0.460	0.154–1.416	0.178
	2a/b	24.54 (6.38–12.03)	0.913	0.455–1.833	0.699
	3a/b	20.69 (4.33–12.20)	0.446	0.244–0.812	0.008
	4a/b	10.41 (1.06–8.33)			
Nodal Status	N+	14.87 (11.15–18.59)	1.293	0.830–2.014	0.256
	N−	274.34 (15.51–33.165)			
Interval primary tumor and LM	Metachronous (>2months)	23.221 (16.22–30.23)	0.687	0.458–1.029	0.069
	Synchronous (≤2 months)	13.86 (9.57–18.15)			
Neoadjuvant chemotherapy	Yes	11.92 (9.52–14.32)	1.764	1.164–2.674	0.008
	No	25.58 (17.79–33.36)			
Type of resection	Minor (3 segments resected or less)	22.08 (16.20–27.96)	0.610	0.404–0.922	0.019
	Major	11.54 (8.27–14.81)			
R0/R1 Resection	R0	20.69 (15.24–26.14)	0.888	0.561–1.405	0.611
	R1	13.35 (9.81–16.90)			
Bloodtransfusion	No	18.48 (14.18–22.78)	0.775	0.495–1.212	0.264
	Yes	16.63 (8.92–24.33)			
Distribution	Bilobar	16.80 (12.31–21.30)	1.018	0.680–1.526	0.931
	Unilobar	19.83 (12.63–27.03)			
Adjuvant chemotherapy	Yes	23.88 (17.47–30.29)	0.564	0.378–0.843	0.005
	No	11.70 (8.40–15.0)			
Tumorsize largest metastasis	≤5 cm	20.57 (15.91–25.43)	0.518	0.341–0.794	0.002
	>5 cm	12.14 (6.86–17.42)			
Tumor cell percentage			0.998	0.989–1.007	0.596
Necrosis percentage			0.999	0.990–1.008	0.817
Fibrosis percentage			0.995	0.983–1.006	0.364
Preoperative CEA			1.140	0.938–1.385	0.189
Postoperative CEA			1.167	0.950–1.439	0.141
Nr. of metastases			2.156	1.227–3.788	0.008
Fong	low risk <3	23.43 (17.34–29.51)	0.543	0.352–0.804	0.005
	high risk ≥3	10. 03 (7.68–12.38)			
Nordlinger	low risk <4	25.49 (17.75–33.25)	0.594	0.395–0.893	0.018
	high risk ≥4	12.13 (9.61–14.67)			
Iwatsuki grade	low risk <3	20.94 (15.05–26.83)	0.705	0.472–1.053	0.117
	high risk ≥3	15.58 (10.45–20.72)			
Mayo Score	low risk <2	18.12 (13.94–22.29)	0.819	0.522–1.285	0.399
	high risk ≥2	17.06 (9.14–24.98)			
Basingstoke index	low risk <10	20.71 (15.56–25.86)	0.551	0.338–0.898	0.016
	high risk ≥10	9.94 (5.95–13.93)			

DFS, disease free survival; HR, hazard ratio; CI, confidence interval; LM, lymph nodes; CEA, carcinoembryonic antigen.

a P values were calculated with the use of log-rank test.

**Figure 5:**
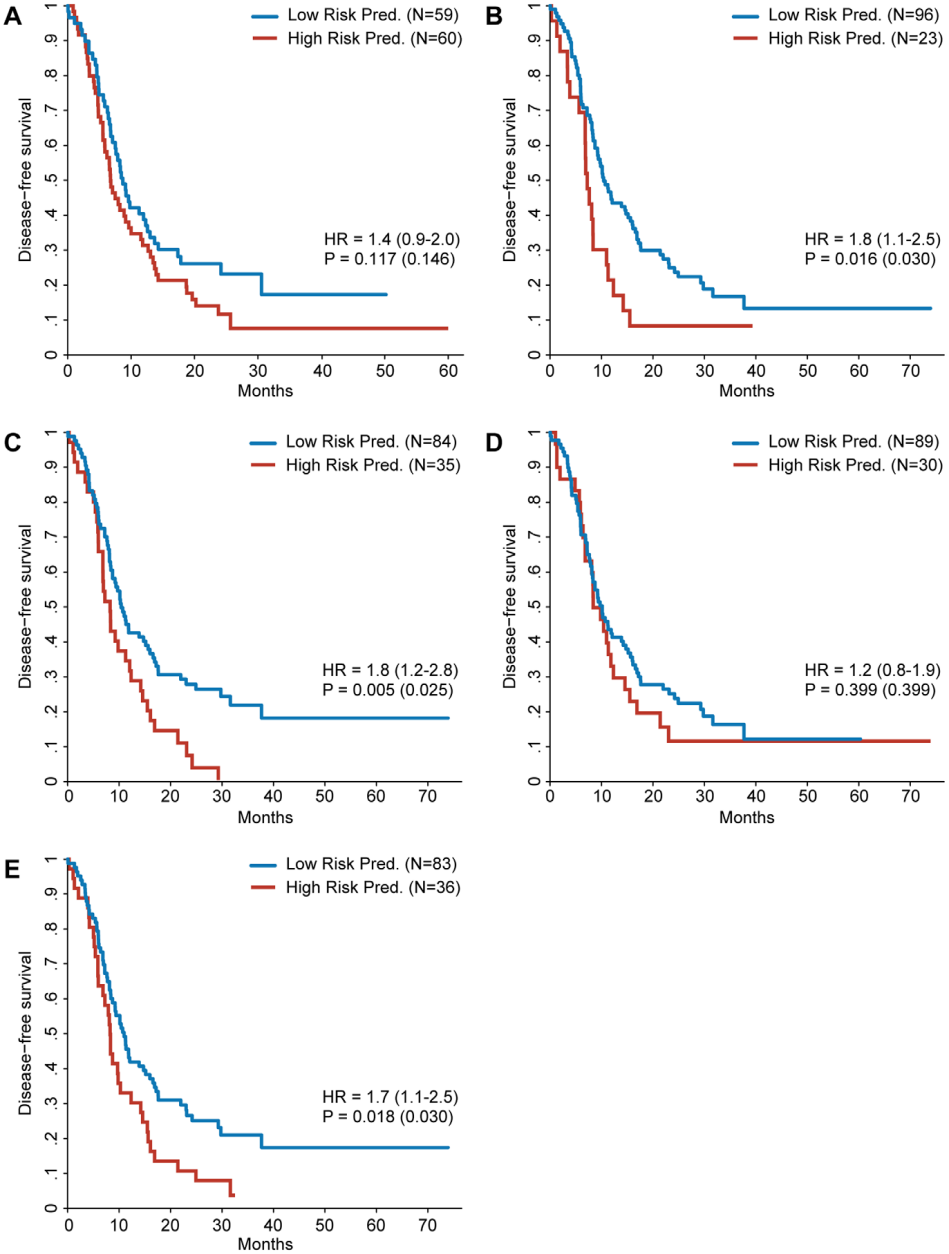
Kaplan–Meier survival analysis for clinical risk predictors. Survival curves based on all 119 patients using known clinical predictors. The hazard ratio of the clinical risk predictor is shown with the 95% confidence interval between brackets. The p value of the log-rank test is shown as well, with the p value adjusted for multiple testing between brackets. **A:** Iwatsuki (high risk ≥3, low risk <3). **B:** Basingstoke (high risk ≥10, low risk <10). **C:** Fong (high risk ≥3, low risk <3). **D:** Mayo (high risk ≥2 low risk <2). **E:** Nordlinger (high risk ≥4, low risk <4).

### Validation of Clinical Risk Scores

The univariate survival analysis results for all clinicopathological factors are depicted in Table 3. In a multivariate Cox regression model, containing the factors that displayed p-values less than 0.1 in univariable analysis, higher stage of the primary tumor (p = 0.006, HR = 1.444, 95% CI = 1.110–1.877), major resection (p = 0.005, HR = 2.190, 95% CI = 1.268–3.784), the number of liver metastases (p = 0.031, HR = 1.142, 95% CI = 1.012–1.289) and the administration of adjuvant chemotherapy (p<0.001, HR = 0.382, 95% CI = 0.237–0.617) were found to be independent risk factors for poor DFS.

All items of the clinical risk scores were documented except for the status of the hepatoduodenal lymph nodes, which made it impossible for the risk score of Zakaria to be higher than 2. Because we did not include patients with extrahepatic disease in this study, the Basingstoke risk score was not complete. Three out of five clinical risk scores predicted DFS accurately in our patients including the Basingstoke, Fong and Nordlinger risk scores (Table 3). Of these, the score by Fong performed best. Kaplan Meier curves for high and low risk predicted patients, based on the different clinical scores, are depicted in Figure 5.

## Discussion

This study was designed to identify and validate a gene expression based classifier that predicts DFS. Unfortunately, we were unable to find a gene signature that could significantly predict DFS in an independent validation set. A gene signature developed using only Paul Brousse patient samples did show a positive trend upon validation. However, in a multivariate Cox regression model, the signature did not prove to be an independent factor for DFS. Instead of reflecting tumor biology, the gene signature appeared to be influenced by a bias in prior administration of chemotherapy, a possibility which should be taken into account when conducting future studies. This view was strengthened both by the absence of predictive power in a gene signature designed in a subset including only Paul Brousse patients receiving neoadjuvant chemotherapy as well as an analysis of differential gene expression between patients treated with neoadjuvant chemotherapy and untreated patients which showed 875 genes differentially expressed. To rule out that the absence of a predictive gene signature was caused by the neoadjuvant treatment bias in the high risk patient group, the signature discovery was repeated using training sets were the neoadjuvant bias was removed as well as analyzing the neoadjuvant treated and untreated patients separately. Similar to earlier results of this study the resulting gene signatures were not predictive of DFS in the validation set indicating that the overrepresentation of neoadjuvant treatment in the high risk patient group does not explain the lack of positive results.

We also tested five known clinical risk scores and found that Basingstoke, Fong and Nordlinger significantly predicted DFS in our patient group. The fact that three out of five scores were predictive is remarkable given the fact that these clinical risk scores (CRS) were developed in an era where the use chemotherapy in primary CRC was rare [14]–[18]. The same five clinical risk scores were recently validated by Reissfelder and colleagues. They found that the Fong and Iwatsuki scores were able to predict disease specific survival in their patients but not Nordlinger and the Basingstoke index [7]. It is remarkable that only the Fong score was predictive in both studies. The non-significant correlation of the Iwatsuki score with DFS could be due to the fact that the highest score could not be calculated, since we did not record the status of the hepatoduodenal lymph nodes. The question remains: why did we not find a signature predicting DFS after resection of colorectal liver metastases? Difficulties in predicting (disease free) survival with gene expression profiling have been reported recently. Lauss et al evaluated the performance of 8 published gene signatures in predicting recurrence in bladder cancer of which none survived the validation [19]. A review evaluating gene signatures developed for predicting survival in lung cancer in 16 studies were all found inadequate for use in clinical practice because of lacking or insufficient validation. In these studies, either the signature did not outperform clinical factors or the authors did not address the influence of any of the clinical factors [20].

We do believe that the design of our study was of sufficient quality to be able find a gene signature for predicting DFS. However, it cannot be excluded that a usable gene signature does exist but was not found due to limiting factors in our study. These potential factors include our definition of high and low risk patients in the signature discovery, the number of patients included in the study especially in light of the heterogeneity of the patient group, the inclusion of patients from only two medical centers, the existence of a prior treatment effect and limits to the sensitivity of microarrays.

Liver metastases are by their nature biased towards a more aggressive subgroup of CRC. It could therefore be speculated that gene expression patterns that characterize rapidly recurring liver metastases are too subtle to be uncovered using the sample size employed in this study. Moreover, recurrence after resection of liver metastases might not be dependent on the characteristics of the liver metastasis itself, but on the presence of micrometastases at the time of liver resection.

Although we cannot exclude the existence of a predictive gene signature, no added benefit of gene expression signatures for the prediction of disease free survival in metastatic colorectal disease could be established based on the results of this study. Finally, the Fong clinical risk score, already validated by Reissfelder et al [7], is the most powerful risk score for predicting DFS of patients with resected CRLM of the five tested risk scores in our study. This clinical risk score should be used for stratification in prospective clinical studies examining the possible benefit of adjuvant therapies in patients undergoing surgery for CRLM.

## Materials and Methods

### Patient Samples

Frozen tumor samples from 148 patients were obtained from the Paul Brousse Hospital in Villejuif, France and the UMC Utrecht in the Netherlands between November 2000 and August 2010. The study protocol was approved by The Medical Ethical Committee (MEC) of the University Medical Center Utrecht as recognized by article 16 of the WMO (Dutch Law on Medical Research with human subjects). Written informed consent was obtained from all patients. Samples were included of patients aged 18 years or older who underwent curative resection for histologically confirmed liver metastases from CRC. Patients with a history of non-colorectal malignancies, extrahepatic disease or macroscopic residual disease (R2) after surgery were excluded. Patients who received local ablative therapy or chemoembolization alone or in combination with resection were excluded. Only specimen were included that were snap frozen in liquid nitrogen within 30 minutes after resection and were stored in −80°C. The amount of stroma, tumor, benign liver cells and necrosis was determined by the two study pathologists (C.G and P.J.vD). Patients whose samples contained benign liver tissue or insufficient tumor cells were excluded from the study. Intraoperative ultrasound of the liver was performed in all patients to assess the size and location of the liver metastases. The size of the dataset was determined by the available patient tumor samples in the two participating institutions which fulfilled all in- and exclusion criteria. Patient-, tumor- and surgical characteristics were extracted from our prospectively collected databases. The definition of synchronous liver metastasis (diagnosis within two months after initial diagnosis) was based on that provided by the US National Cancer Institute.

### Follow-up

All patients received standard follow up with spiral CT of the abdomen and chest every 3 months to monitor recurrences. Disease free survival was defined as the time from resection to the time of the first sign of recurrence on CT scanning. All patients were censored at the time of death or the last follow-up. Survival time was determined using the Kaplan-Meier survival function.

### Gene Expression Profiling

#### RNA isolation

Total RNA was isolated from individual tissue samples using Trizol reagent (Invitrogen) following the manufacturer’s protocol. RNA was purified using the RNeasy mini-kit (Qiagen) and was subjected to DNase treatment using the Qiagen DNA-free kit. The yield and quality of total RNA was checked by spectrophotometry and by the Agilent Bioanalyzer (Agilent). Thirteen samples were excluded on the basis of the RNA yield and cRNA yield (RNA integrity number [RIN] <6). Eight samples were excluded due to amplification failures, and 8 more samples did not meet the labeling criteria, resulting in data from 119 samples.

#### cRNA synthesis and fluorescent labeling

All amplification and labelling procedures were performed in 96 wells plates (4titude, Bioke) on a customized Sciclone ALH 3000 Workstation (Caliper LifeSciences), with a PCR PTC-200 (Bio-Rad Laboratories), SpectraMax 190 spectrophotometer (Molecular Devices), and a magnetic bead-locator (Beckman). cRNA products were purified and concentrated with RNAClean (Agencourt, Beckman) according to manufacturer’s protocol. mRNA was amplified by in vitro transcription using an anchored primer and T7 RNA polymerase on 1 µg of total RNA. First a double stranded cDNA template was generated including the T7 promoter. Next, this template was used for in vitro transcription with the T7 megascript kit (Ambion) to generate cRNA. During the in vitro transcription, 5-(3-aminoallyl)-UTP (Ambion) was incorporated into the single stranded cRNA. Samples with a yield less than 2000 ng or with small cRNA fragments (median less than 500 nt) were not used. Cy3 or cy5 fluorophores (GE Healthcare) were coupled to cRNA. We applied total RNA and cRNA quality control criteria in accordance with the Tumor Analysis Best Practices Working Group [21]. The yield and label incorporation of the cy-labeled cRNA was checked using spectrophotometry. Only samples with between 1.5% and 3% Cy-incorporation were included. Before hybridization, 300–1000 ng of Cy-labeled cRNA from one biopsy was mixed with an equal amount of reverse color Cy-labeled material from the reference sample.

#### Microarray hybridization

For each sample, two expression profiles in dye-swap experiments were generated. The samples were compared against a commercial reference (Universal Human Reference RNA catalog #740000, Stratagene). The Human Array-Ready Oligo set (version 2.0) was purchased from Qiagen and spotted on Codelink slides (GE Healthcare) in a dust filtered and humidity controlled clean room. The microarrays contained 70-mer oligo-nucleotides representing 21.329 human genes and expressed sequence tags (ESTs), as well as 3871 additional spots for control purposes. Gene annotations were updated by BLAST analysis of all feature sequences using ENSEMBL build 55. Arrays were hybridized on a Tecan HS4800PRO hybridization station, using the protocol described previously [22]. Hybridized slides were scanned on an Agilent scanner (G2565BA) at 100% laser power and 60–90% PMT. After automatic data extraction using Imagene 8.0.1 (BioDiscovery), printtip Loess normalization was performed on mean spot intensities [23]. Dye bias was corrected based on a within-set estimate [24].

#### Data accessibility

In accordance with proposed MIAME (Minimum information about a microarray experiment) standards, primary and processed data as well as protocols were deposited in Array Express (http://www.ebi.ac.uk/microarray-as/aer) under accession number E-TABM-1112.

### Identification of a Recurrence Signature

The cohort was randomly divided in a training set (n = 75) and a validation set (n = 44). The latter was not involved in gene selection to avoid a selection bias. For the purposes of discovering the gene signature, patients were initially divided in a high risk and a low risk group. High risk patients were defined as those with recurrence within 1 year (Figure 2). This threshold was based on the observation that a DFS <1 year is predictive of adverse overall survival as described by Fong et al [14]. A division based on DFS ≤6 months (high risk) and DFS >2 years (low risk) was also applied (Figure 2B). Using the training set, genes were ranked based on three different metrics (signal-to-noise-ratio, t-test statistic and Cox proportional hazard ratio). This ranking was done using a multiple sampling approach selecting 2/3 of the samples in each iteration. The 75 top ranked genes were used to predict the risk class of the samples in the remaining 1/3 of samples using nearest mean classification [9] and leave-one-out cross validation (LOOCV). Using these predictions a combined area under the curve for 1000 iterations was calculated giving an indication of the aggregated predictive power of the 75 gene signatures, where a value significantly above 0.5 points to true predictive power. The ranking of the genes were averaged over all 1000 iterations [25]. From the resulting ranked list, the gene signature with the strongest prognostic power (measured as overall accuracy of prediction) was determined using nearest mean classification and LOOCV starting from the best ranked gene and subsequently adding the next highest ranked gene in each iteration (forward selection) [9]. An independent measure of the predictive power was obtained by using the resulting gene signature to predict the risk class of the samples in the validation set (nearest mean, LOOCV). Kaplan-Meier analyses were used to estimate DFS and survival curves for the two predicted risk classes were compared using the Mantel-Cox log-rank test. A power analysis for the log-rank test was done using the PS program [26]. Functional gene set enrichment analysis was performed using the Babelomics 4.2 web-based analysis suite including all the databases available for the enrichment analysis [27].

### Analysis of Differential Gene Expression

Gene expression in patients treated with neoadjuvant chemotherapy was compared to expression in untreated patients using ANOVA [28]. In a fixed effect analysis, sample, array and dye effects were modelled. P values were determined by a permutation F2-test in which residuals were shuffled 5000 times globally.

### Clinical Risk Scores

A univariate Cox proportional hazards regression model was used to estimate the hazard ratios of five clinical risk scores which were calculated for each patient [14]–[18] A multivariate analysis was also performed entering the factors with p values below 0.1 in the univariate analysis.

### Statistical Testing and Software

All statistical tests were two-sided and statistical significance was assumed for p values less than 0.05. Where applicable, p values were adjusted for their false discovery rate using the Benjamini-Hochberg method [29]. Statistical analyses were done in R 2.7.0 with additional Bioconductor packages and SPSS for Windows version 15.0 (SPSS, Chicago, Illinois, USA).

## Supporting Information

Figure S1 Power of the log-rank test. The statistical power of the log-rank test as a function of the hazard ratio of the gene signature prediction in the validation set.
(TIF)

Table S1 Patient- and tumor characteristics of the in- and excluded patients.^a^
(DOC)

Table S2 Univariate Cox regression analysis for the signature genes.^a^
(DOC)

Table S3 Patient- and tumor characteristics per center.^a^
(DOC)

Table S4 Genes differentially expressed between patients treated with neoadjuvant chemotherapy and untreated patients.
(DOC)

Table S5 Signature performances for predicting DFS as a dichotomous outcome.^a^
(DOC)
