# Supplementary figures and images for: Exploring Gene Expression Signatures for Predicting Disease Free Survival after Resection of Colorectal Cancer Liver Metastases
Source: PLoS One. 2012 Nov 21;7(11):e49442. doi: 10.1371/journal.pone.0049442 (PMC3504021; doi:10.1371/journal.pone.0049442)

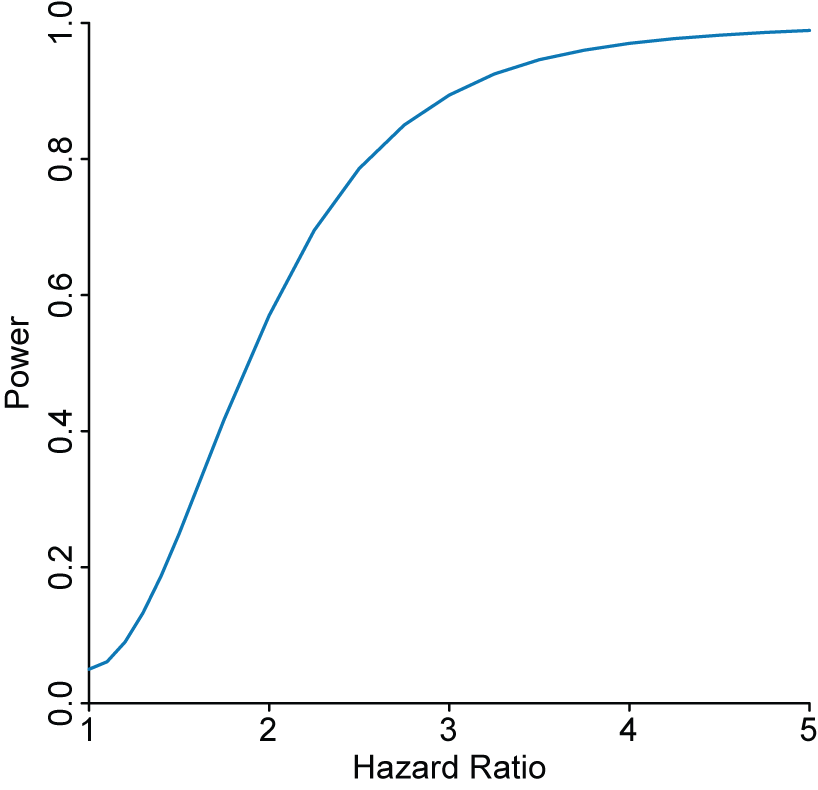

Supplement: Figure S1 — Power of the log-rank test. The statistical power of the log-rank test as a function of the hazard ratio of the gene signature prediction in the validation set. (TIF) [file pone.0049442.s001.tif]
