# Supplementary material for: Exploring Gene Expression Signatures for Predicting Disease Free Survival after Resection of Colorectal Cancer Liver Metastases
Source: PLoS One. 2012 Nov 21;7(11):e49442. doi: 10.1371/journal.pone.0049442 (PMC3504021; doi:10.1371/journal.pone.0049442)
Supplement: Table S1 — Patient- and tumor characteristics of the in- and excluded patients.a (DOC) [file pone.0049442.s002.doc]

**Table S1: Patient- and tumor characteristics of the in- and excluded patients^a^**

| **Category** | **Subcategory** | **Included**  **patients** | **Excluded patients** | **Total** | **P value^b^** |
| --- | --- | --- | --- | --- | --- |
| Total number of patients |  | **119** | **29** | **148** |  |
| Sex | Male | 77 (64.7%) | 16 (55.2%) | 93 (62.8%) | 0.342 |
|  | Female | 42 (35.5%) | 13 (44.8%) | 55 (37.1%) |  |
| Age (Mean; SD) |  | 61.4 (11.43) | 63.55 (9.72) | 61.82 (11.12) | 0.348 |
| Location of primary tumor | Rectum | 30 (25.2%) | 10 (34.5%) | 40 (27.2%) | 0.316 |
|  | Colon | 89 (74.8%) | 19 (65.5%) | 108 (73.0%) |  |
| Differentiation primary tumor | Good | 16 (13.4%) | 6 (20.7%) | 22 (14.9%) | 0.084 |
|  | Moderate | 86 (72.3%) | 22 (75.9%) | 108 (73.0%) |  |
|  | Poor | 17 (13.4%) | 1 (3.4%) | 18 (12.2%) |  |
| Nodal Status | N+ | 66 (55.5%) | 16 (55.2%) | 82 (55.4%) | 0.489 |
|  | N- | 40 (33.6) | 13 (44.8%) | 53 (35.8%) |  |
|  | Missing | 13 (10.9%) |  | 13 (8.7%) |  |
| Interval primary tumor and LM | Metachronous (>2 months) | 61 (51.3%) | 12 (41.4%) | 73 (49.4%) | 0.342 |
|  | Synchronous (≤2 months) | 58 (48.7%) | 17 (58.6%) | 75 (50.6%) |  |
| Neoadjuvant chemotherapy | Yes | 64 (53.8%) | 13 (44.8%) | 77 (52.1%) | 0.893 |
|  | No | 55 (46.2%) | 16 (55.2%) | 71 (47.9%) |  |
| Type of resection | Minor (≤3 segments resected) | 76 (63.9%) | 18 (62.1%) | 94 (63.5%) | 0.857 |
|  | Major | 43 (36.1%) | 11 (37.9%) | 54 (36.5%) |  |
| R0/R1 Resection | R0 | 88 (73.9%) | 21 (72.4%) | 109 (73.6%) | 0.756 |
|  | R1 | 29 (24.4%) | 8 (27.6%) | 37 (25.0%) |  |
|  | Missing | 2 (1.7%) |  | 2 (1.4%) |  |
| Bloodtransfusion | No | 86 (72.3%) | 17 (58.6%) | 103 (69.6%) | 0.119 |
|  | Yes | 31 (26.1%) | 12 (41.4%) | 43 (29.1%) |  |
|  | Missing | 2 (1.7%) |  | 2 (1.4%) |  |
| Distribution | Bilobar | 50 (42.0%) | 7 (24.1%) | 57 (38.5%) | 0.076 |
|  | Unilobar | 68 (57.1%) | 22 (75.9%) | 90 (60.8%) |  |
|  | Missing | 1 (0.8%) |  | 1 (0.7%) |  |
| Mean number of LM/Patient |  | 2.58 (2.43) | 2.41 (1.59) | 2.55 (2.28) | 0.725 |
| Tumorsize biggest metastases (cm) |  | 4.93 (3.17) | 4.03 (2.28) | 4.75 (3.02) | 0.160 |
| Preoperative CEA (Mean, SD) |  | 79.98 (146.36) | 27.8 (81.26) | 72.67 (139.99) | 0.175 |
| Postoperative CEA (Mean, SD) |  | 12.57 (50.27) | 2.39 (2.15) | 10.51 (45.06) | 0.219 |
| Adjuvant chemotherapy | Yes | 68 (57.1%) | 14 (48.3%) | 82 (55.4%) | 0.390 |
|  | No | 51 (42.9%) | 15 (51.7%) | 66 (44.6%) |  |

LM, lymph nodes; CEA, carcinoembryonic antigen

^a^ Percentages may not total 100 because of rounding.

^b^ *P* values were calculated with the use of Mann-Whitney test for continuous variables and Fisher’s exact test for categorical variables.
