# Supplementary material for: Exploring Gene Expression Signatures for Predicting Disease Free Survival after Resection of Colorectal Cancer Liver Metastases
Source: PLoS One. 2012 Nov 21;7(11):e49442. doi: 10.1371/journal.pone.0049442 (PMC3504021; doi:10.1371/journal.pone.0049442)
Supplement: Table S2 — Univariate Cox regression analysis for the signature genes.a (DOC) [file pone.0049442.s003.doc]

**Table S2: Univariate Cox regression analysis for the signature genes^a^**

| **Gene Name** | **Training** | | | **Validation** | | |
| --- | --- | --- | --- | --- | --- | --- |
|  | **P value^b^** | **HR** | **95% CI** | **P value^b^** | **HR** | **95% CI** |
| CCDC85A | 0.087 | 0.17 | 0.02-1.29 | 0.623 | 0.05 | 0-1.65 |
| MYNN | <0.001 | 169.07 | 17.94-1593.2 | 0.784 | 0.17 | 0-8.37 |
| RP11-347C12.2 | 0.001 | 27.06 | 4.75-154.14 | 0.951 | 1.11 | 0.04-33.16 |
| CPLX1 | 0.017 | 0.05 | 0.01-0.51 | 0.784 | 2.73 | 0.35-21.28 |
| hsa-mir-103-2 | 0.029 | 9.27 | 1.39-61.82 | 0.951 | 1.16 | 0.06-21.75 |
| FRMD6 | 0.017 | 8.59 | 1.63-45.3 | 0.737 | 0.21 | 0.02-2.59 |
| genomic:chrX-142692034-142692102 | 0.010 | 10.68 | 2.39-47.66 | 0.951 | 0.55 | 0.04-8.25 |
| OR5P2 | 0.012 | 0.1 | 0.02-0.48 | 0.951 | 1.21 | 0.05-30.29 |
| C6orf141 | 0.030 | 0.08 | 0.01-0.72 | 0.935 | 0.37 | 0.01-11.06 |
| FAM174B | 0.012 | 20.91 | 2.82-155.14 | 0.737 | 2.68 | 0.6-12.03 |
| Unknown^c^ | 0.021 | 13.38 | 1.67-107.32 | 0.935 | 0.45 | 0.02-9.33 |
| RIPK4 | 0.012 | 0.07 | 0.01-0.44 | 0.784 | 0.15 | 0-5.53 |
| GPR143 | 0.044 | 0.12 | 0.02-0.89 | 0.951 | 1.63 | 0.11-23.58 |
| ITSN1 | 0.012 | 0.04 | 0-0.38 | 0.458 | 127.3 | 1.96-8283.46 |
| MAPKAPK2 | 0.013 | 0.03 | 0-0.38 | 0.623 | 63.68 | 0.79-5107.74 |
| KIAA0562 | 0.012 | 10.83 | 2.01-58.17 | 0.784 | 3.56 | 0.16-77.07 |
| PARN | 0.065 | 9.5 | 0.92-98.25 | 0.784 | 0.27 | 0.01-7.13 |
| OTUD5 | 0.012 | 31.23 | 2.66-366.01 | 0.951 | 1.22 | 0.05-32.62 |
| ZNF134 | 0.077 | 6.58 | 0.84-51.79 | 0.690 | 11.99 | 0.45-319.46 |
| BAT2 | <0.001 | 6.77 | 3.06-14.98 | 0.951 | 0.93 | 0.28-3.13 |

HR, hazard ratio; CI, confidence interval; DFS, disease free survival

^a^ Discovered using all samples in training set defining high-risk as DFS ≤ 1 year and low-risk as DFS > 1 year.

^b^ Adjusted for multiple testing with Benjamini-Hochberg.

^c^ Probe sequence cannot be confidently mapped to a transcript or genomic location.
