# Supplementary material for: Exploring Gene Expression Signatures for Predicting Disease Free Survival after Resection of Colorectal Cancer Liver Metastases
Source: PLoS One. 2012 Nov 21;7(11):e49442. doi: 10.1371/journal.pone.0049442 (PMC3504021; doi:10.1371/journal.pone.0049442)
Supplement: Table S3 — Patient- and tumor characteristics per center.a (DOC) [file pone.0049442.s004.doc]

**Table S3: Patient- and tumor characteristics per center^a^**

| **Category** | **Subcategory** | **Paul Brousse** | **UMC Utrecht** | **Total** | **P value^b^** |
| --- | --- | --- | --- | --- | --- |
| Total number of patients |  | 73 | 46 | 119 |  |
| Sex | Male | 46 (63.0%) | 31 (67.4%) | 77 (64.7%) | 0.627 |
|  | Female | 27 (37.0%) | 15 (32.6%) | 42 (35.3%) |  |
| Age (Mean, SD) |  | 60.98(11.75) | 62.04 (11) | 61.4 (11.43) | 0.621 |
| Location of primary tumor | Rectum | 16 (21.9%) | 14 (30.4%) | 30 (25.2%) | 0.299 |
|  | Colon | 57 (78.1%) | 32 (69.6%) | 89 (74.8%) |  |
| Differentiation primary tumor | Good | 12 (16.4%) | 5 (10.9%) | 16 (13.4%) | 0.656 |
|  | Moderate | 50 (68.5%) | 36 (78.3%) | 86 (72.3%) |  |
|  | Poor | 11 (15.1%) | 5 (10.9%) | 16 (13.4%) |  |
| Nodal Status | N+ | 41 (56.2%) | 25 (54.3%) | 66 (55.5%) | 0.143 |
|  | N- | 19 (26.0%) | 21 (45.7%) | 40 (33.6) |  |
|  | Missing | 13 (9.6%) |  | 13 (10.9%) |  |
| Interval primary tumor and LM | Metachronous (>2 months) | 32 (43.8%) | 29 (63.0%) | 61 (51.3%) | 0.043 |
|  | Synchronous (≤2 months) | 41 (56.2%) | 17 (37.0%) | 58 (48.7%) |  |
| Neoadjuvant chemotherapy | Yes | 54 (74.0%) | 36 (78.3%) | 64 (53.8%) | <0.001 |
|  | No | 19 (26.0%) | 10 (21.7%) | 55 (46.2%) |  |
| Type of resection | Minor (≤3 segments resected) | 40 (54.8%) | 36 (78.3%) | 76 (63.9%) | 0.014 |
|  | Major | 33 (45.2%) | 10 (21.7%) | 43 (36.1%) |  |
| R0/R1 Resection | R0 | 45 (61.6%) | 43 (93.5%) | 88 (73.9%) | 0.001 |
|  | R1 | 26 (35.6%) | 3 (6.5%) | 29 (24.4%) |  |
|  | Missing | 2 (2.7%) |  | 2 (1.7%) |  |
| Bloodtransfusion | No | 45 (61.6%) | 41 (89.1%) | 86 (72.3%) | 0.004 |
|  | Yes | 26 (35.6%) | 5 (10.9%) | 31 (26.1%) |  |
|  | Missing | 2 (2.7%) |  | 2 (1.7%) |  |
| Distribution | Bilobar | 37 (50.7%) | 31 (67.4%) | 68 (57.1%) | 0.088 |
|  | Unilobar | 35 (47.9%) | 15 (32.6%) | 50 (42.4%) |  |
|  | Missing | 1 (1.4%) |  | 1 (0.8%) |  |
| Mean number of LM/Patient |  | 2.86 (2.75) | 2.13 (1.73) | 2.58 (2.43) | 0.120 |
| Tumorsize biggest metastases (cm) |  | 4.93 (3.17) | 4.93 (3.19) | 4.93 (3.17) | 0.014 |
| Tumor cell percentage (Mean, SD) |  | 39.70 (17.43) | 53.04 (28.18) | 45.18 (23.62) | 0.004 |
| Necrosis percentage (Mean, SD) |  | 20.82 (15.52) | 16.96 (19.73) | 19.33 (17.30) | 0.014 |
| Fibrosis percentage (Mean, SD) |  | 24.41 (15.33) | 16.96 (19.73) | 19.66 (17.25) | 0.177 |
| Preoperative CEA (Mean, SD) |  | 86.39 (166.14) | 67.36 (97.20) | 79.98 (146.36) | 0.533 |
| Postoperative CEA (Mean, SD) |  | 15.11 (62.48) | 8.67 (21.19) | 12.57 (50.27) | 0.572 |
| Adjuvant chemotherapy | Yes | 18 (24.7%) | 33 (71.7%) | 51 (42.9%) | <0.001 |
|  | No | 55 (75.3%) | 13 (28.3%) | 68 (57.1%) |  |

LM, lymph nodes; CEA, carcinoembryonic antigen

^a^ Percentages may not total 100 because of rounding.

^b^ P values were calculated with the use of Mann-Whitney test for continuous variables and Fisher’s exact test for categorical variables.
