# Supplementary material for: Exploring Gene Expression Signatures for Predicting Disease Free Survival after Resection of Colorectal Cancer Liver Metastases
Source: PLoS One. 2012 Nov 21;7(11):e49442. doi: 10.1371/journal.pone.0049442 (PMC3504021; doi:10.1371/journal.pone.0049442)
Supplement: Table S4 — Genes differentially expressed between patients treated with neoadjuvant chemotherapy and untreated patients. (DOC) [file pone.0049442.s005.doc]

**Table S4: Genes differentially expressed between patients treated with neoadjuvant chemotherapy and untreated patients**

| **Gene Name** | **P value^a^** | **M value** |
| --- | --- | --- |
| MGP | < 0.001 | 0.855538 |
| REG1A | < 0.001 | 0.674406 |
| C1QC | < 0.001 | 0.639766 |
| IFI27 | < 0.001 | 0.604821 |
| TYROBP | < 0.001 | 0.589622 |
| TMEM176B | < 0.001 | 0.544941 |
| FCER1G | < 0.001 | 0.540644 |
| DEFA5 | < 0.001 | 0.52404 |
| DCN | < 0.001 | 0.523328 |
| HLA-DRA | < 0.001 | 0.506665 |
| HLA-DPA1 | < 0.001 | 0.435674 |
| HLA-DPB1 | < 0.001 | 0.434246 |
| CCL2 | < 0.001 | 0.43289 |
| CD74 | < 0.001 | 0.422907 |
| COLEC11 | < 0.001 | 0.419372 |
| CD52 | < 0.001 | 0.407947 |
| CST3 | < 0.001 | 0.402686 |
| EFEMP1 | < 0.001 | 0.400427 |
| CYP1B1 | < 0.001 | 0.391459 |
| SLA | < 0.001 | 0.38729 |
| LAPTM5 | < 0.001 | 0.383373 |
| C1QA | < 0.001 | 0.380021 |
| PLXDC2 | < 0.001 | 0.373442 |
| LIPA | < 0.001 | 0.367993 |
| CTSK | < 0.001 | 0.360842 |
| TAGLN | < 0.001 | 0.360381 |
| EGR4 | < 0.001 | 0.353639 |
| MATN4 | < 0.001 | 0.347637 |
| S100A9 | < 0.001 | 0.344839 |
| LXN | < 0.001 | 0.343703 |
| SLC15A3 | < 0.001 | 0.340785 |
| CYBRD1 | < 0.001 | 0.335374 |
| CMTM6 | < 0.001 | 0.334918 |
| BCAT1 | < 0.001 | 0.330798 |
| SPARCL1 | < 0.001 | 0.33079 |
| HLA-DQA1 | < 0.001 | 0.330579 |
| ENY2 | < 0.001 | 0.330529 |
| GPR52 | < 0.001 | 0.325175 |
| C8orf83 | < 0.001 | 0.316285 |
| SNRPB2 | < 0.001 | 0.315692 |
| CCL21 | < 0.001 | 0.315508 |
| CCL19 | < 0.001 | 0.309933 |
| TCEAL3 | < 0.001 | 0.307393 |
| DUSP4 | < 0.001 | 0.306765 |
| BMPR1A | < 0.001 | 0.3038 |
| AC012379.7 | < 0.001 | 0.295964 |
| HLA-DMB | < 0.001 | 0.295288 |
| CEBPD | < 0.001 | 0.293497 |
| MS4A7 | < 0.001 | 0.292674 |
| BLOC1S2 | < 0.001 | 0.29234 |
| MGST3 | < 0.001 | 0.291837 |
| COX7A1 | < 0.001 | 0.29112 |
| TRAM1 | < 0.001 | 0.286865 |
| FABP4 | < 0.001 | 0.285985 |
| genomic:14+89018975-89019043 | < 0.001 | 0.285678 |
| LMCD1 | < 0.001 | 0.283035 |
| FCGR1A | < 0.001 | 0.275731 |
| NPC2 | < 0.001 | 0.271777 |
| HLA-DOA | < 0.001 | 0.270233 |
| PLTP | < 0.001 | 0.268902 |
| CANX | < 0.001 | 0.264815 |
| CASC4 | < 0.001 | 0.264445 |
| RB1CC1 | < 0.001 | 0.256793 |
| GMFG | < 0.001 | 0.256392 |
| HK1 | < 0.001 | 0.251146 |
| UQCRB | < 0.001 | 0.248705 |
| CD48 | < 0.001 | 0.244452 |
| TIMP3 | < 0.001 | 0.244286 |
| RBP2 | < 0.001 | 0.243123 |
| GPX1 | < 0.001 | 0.241845 |
| C1orf54 | < 0.001 | 0.241546 |
| BCL2L10 | < 0.001 | 0.24105 |
| AMPD1 | < 0.001 | 0.238858 |
| LY96 | < 0.001 | 0.237905 |
| DARC | < 0.001 | 0.237157 |
| DACT1 | < 0.001 | 0.237118 |
| NA | < 0.001 | 0.236671 |
| EMP1 | < 0.001 | 0.236582 |
| LHFPL2 | < 0.001 | 0.236535 |
| DAD1 | < 0.001 | 0.234797 |
| OPCML | < 0.001 | 0.233528 |
| HAVCR2 | < 0.001 | 0.233125 |
| CCDC90B | < 0.001 | 0.232219 |
| TGFBR2 | < 0.001 | 0.231656 |
| APLP2 | < 0.001 | 0.231171 |
| ATP6V0E | < 0.001 | 0.228941 |
| TNFAIP8 | < 0.001 | 0.228699 |
| FMO4 | < 0.001 | 0.228325 |
| MAFB | < 0.001 | 0.226949 |
| MRPS16 | < 0.001 | 0.225745 |
| MYL6 | < 0.001 | 0.225721 |
| RPS23 | < 0.001 | 0.224501 |
| GLRX | < 0.001 | 0.220206 |
| TMED3 | < 0.001 | 0.218812 |
| MTDH | < 0.001 | 0.218269 |
| COX7 | < 0.001 | 0.217932 |
| FBXL5 | < 0.001 | 0.216259 |
| MYADM | < 0.001 | 0.213536 |
| COX8A | < 0.001 | 0.213534 |
| NCF1C | < 0.001 | 0.213385 |
| ARL6IP5 | < 0.001 | 0.212502 |
| C18orf32 | < 0.001 | 0.212458 |
| PTRH2 | < 0.001 | 0.211008 |
| RNASEH2C | < 0.001 | 0.210844 |
| LSMD1 | < 0.001 | 0.207684 |
| CETN2 | < 0.001 | 0.206773 |
| MAF1 | < 0.001 | 0.206624 |
| LHFP | < 0.001 | 0.206429 |
| FAM82B | < 0.001 | 0.206348 |
| AP001453.6-1 | < 0.001 | 0.20601 |
| EHD4 | < 0.001 | 0.205748 |
| TMEM98 | < 0.001 | 0.205489 |
| SNAP23 | < 0.001 | 0.205263 |
| ZNF615 | < 0.001 | 0.204742 |
| MRPL1 | < 0.001 | 0.204568 |
| AC012467.9-1 | < 0.001 | 0.204473 |
| STMN2 | < 0.001 | 0.204399 |
| RABAC1 | < 0.001 | 0.204014 |
| SLCO2A1 | < 0.001 | 0.203402 |
| SERINC1 | < 0.001 | 0.203259 |
| LY6G6E | < 0.001 | 0.20314 |
| MS4A4A | < 0.001 | 0.202261 |
| LTBP2 | < 0.001 | 0.202043 |
| AC174470.1-1 | < 0.001 | 0.201953 |
| RPS27L | < 0.001 | 0.200393 |
| TNS1 | < 0.001 | 0.200238 |
| DDX50 | < 0.001 | 0.200172 |
| AC023024.6 | < 0.001 | 0.199804 |
| TMSB4Y | < 0.001 | 0.199553 |
| PLEKHQ1 | < 0.001 | 0.199008 |
| CORO1C | < 0.001 | 0.198696 |
| SAR1A | < 0.001 | 0.198361 |
| C8orf40 | < 0.001 | 0.196555 |
| genomic:X-99920144-99920212 | < 0.001 | 0.195571 |
| CD58 | < 0.001 | 0.193634 |
| CPM | < 0.001 | 0.193155 |
| FEM1B | < 0.001 | 0.192746 |
| AP4E1 | < 0.001 | 0.19264 |
| SLC35B2 | < 0.001 | 0.191996 |
| SKP1A | < 0.001 | 0.191538 |
| RAF1 | < 0.001 | 0.191529 |
| HSBP1 | < 0.001 | 0.191362 |
| TSPO | < 0.001 | 0.189687 |
| DYNLT1 | < 0.001 | 0.188951 |
| GMFB | < 0.001 | 0.188915 |
| C11orf10 | < 0.001 | 0.187973 |
| NSMCE1 | < 0.001 | 0.187659 |
| GALNT1 | < 0.001 | 0.187391 |
| RAB6A | < 0.001 | 0.186032 |
| CASS4 | < 0.001 | 0.185644 |
| ATP6AP2 | < 0.001 | 0.185174 |
| GPBP1 | < 0.001 | 0.184014 |
| NOSIP | < 0.001 | 0.183894 |
| ARL3 | < 0.001 | 0.183728 |
| SRP14 | < 0.001 | 0.183463 |
| NDUFS4 | < 0.001 | 0.183354 |
| NDUFA2 | < 0.001 | 0.182962 |
| GLIPR1 | < 0.001 | 0.182689 |
| VPS4B | < 0.001 | 0.182535 |
| UBLCP1 | < 0.001 | 0.181752 |
| ATP5H | < 0.001 | 0.181112 |
| KPNA4 | < 0.001 | 0.179846 |
| TNFSF12 | < 0.001 | 0.1793 |
| PIG-Y | < 0.001 | 0.178836 |
| ABLIM3 | < 0.001 | 0.178014 |
| SPCS3 | < 0.001 | 0.176735 |
| TMEM126A | < 0.001 | 0.176676 |
| GPR34 | < 0.001 | 0.175055 |
| CD59 | < 0.001 | 0.17474 |
| RAB33B | < 0.001 | 0.174147 |
| ANKRD40 | < 0.001 | 0.173975 |
| EXOSC4 | < 0.001 | 0.173958 |
| CNN2 | < 0.001 | 0.173753 |
| UBE2B | < 0.001 | 0.173606 |
| PJA2 | < 0.001 | 0.172364 |
| RYK | < 0.001 | 0.171211 |
| PDCD10 | < 0.001 | 0.170779 |
| MFSD3 | < 0.001 | 0.17019 |
| SIRT1 | < 0.001 | 0.169995 |
| TIMM8B | < 0.001 | 0.169406 |
| SLC10A3 | < 0.001 | 0.169181 |
| C19orf63 | < 0.001 | 0.168829 |
| SSPN | < 0.001 | 0.168148 |
| SCAMP1 | < 0.001 | 0.167932 |
| GRAMD3 | < 0.001 | 0.167731 |
| PAIP2 | < 0.001 | 0.167588 |
| SCYL3 | < 0.001 | 0.167258 |
| HSPB2 | < 0.001 | 0.165055 |
| GPR18 | < 0.001 | 0.163811 |
| ARHGDIA | < 0.001 | 0.163608 |
| SEC11B | < 0.001 | 0.16275 |
| NDUFB1 | < 0.001 | 0.162722 |
| ARHGAP15 | < 0.001 | 0.162692 |
| PAFAH1B2 | < 0.001 | 0.162241 |
| TOB2 | < 0.001 | 0.161953 |
| PCSK5 | < 0.001 | 0.161807 |
| CORO1B | < 0.001 | 0.160134 |
| TBCB | < 0.001 | 0.157196 |
| DNAJB12 | < 0.001 | 0.157094 |
| BCL7C | < 0.001 | 0.156201 |
| COQ10B | < 0.001 | 0.155564 |
| NDUFA13 | < 0.001 | 0.155177 |
| DHX29 | < 0.001 | 0.152039 |
| LMOD1 | < 0.001 | 0.148742 |
| EDIL3 | < 0.001 | 0.147744 |
| NA | < 0.001 | 0.146649 |
| DPH3 | < 0.001 | 0.146078 |
| MED31 | < 0.001 | 0.145546 |
| ANAPC13 | < 0.001 | 0.145281 |
| TCEB1 | < 0.001 | 0.144743 |
| CCNJ | < 0.001 | 0.143103 |
| ACTR10 | < 0.001 | 0.14252 |
| MAS1 | < 0.001 | 0.141937 |
| C6orf72 | < 0.001 | 0.140791 |
| PURA | < 0.001 | 0.140079 |
| HPS6 | < 0.001 | 0.13969 |
| SNX11 | < 0.001 | 0.138967 |
| THAP1 | < 0.001 | 0.137101 |
| RRAS | < 0.001 | 0.135032 |
| UBAP2L | < 0.001 | -0.131032 |
| genomic:18-74412239-74412307 | < 0.001 | -0.13125 |
| RBMS3 | < 0.001 | -0.137437 |
| AC114982.2-2 | < 0.001 | -0.138216 |
| genomic:7+123085445-123085481 | < 0.001 | -0.141469 |
| GOLGA4 | < 0.001 | -0.143045 |
| genomic:19-1574409-1574477 | < 0.001 | -0.144057 |
| NCAPD2 | < 0.001 | -0.145632 |
| genomic:19+41455787-41455855 | < 0.001 | -0.148082 |
| SRRM2 | < 0.001 | -0.148768 |
| UBE2I | < 0.001 | -0.150461 |
| AMH | < 0.001 | -0.155392 |
| FAF1 | < 0.001 | -0.157479 |
| MSH3 | < 0.001 | -0.158945 |
| NNT | < 0.001 | -0.159405 |
| UBAP2 | < 0.001 | -0.159827 |
| IPO9 | < 0.001 | -0.160119 |
| NOP2 | < 0.001 | -0.160825 |
| SLC30A5 | < 0.001 | -0.161221 |
| genomic:1+232964184-232964252 | < 0.001 | -0.161632 |
| EXOC5 | < 0.001 | -0.16179 |
| genomic:14+92537614-92537683 | < 0.001 | -0.162694 |
| FAM175A | < 0.001 | -0.162893 |
| D2HGDH | < 0.001 | -0.164872 |
| DARS | < 0.001 | -0.164999 |
| ATP13A1 | < 0.001 | -0.165995 |
| SPTLC3 | < 0.001 | -0.166729 |
| PDAP1 | < 0.001 | -0.167065 |
| genomic:1+21357344-21357412 | < 0.001 | -0.167584 |
| RBP5 | < 0.001 | -0.167774 |
| DFNB31 | < 0.001 | -0.16792 |
| USP10 | < 0.001 | -0.167965 |
| ACAD11 | < 0.001 | -0.168574 |
| SGTA | < 0.001 | -0.17013 |
| SNAPC1 | < 0.001 | -0.170296 |
| AGBL1 | < 0.001 | -0.170514 |
| ZNF273 | < 0.001 | -0.171191 |
| SDCCAG8 | < 0.001 | -0.172127 |
| OCIAD1 | < 0.001 | -0.173122 |
| SMG1 | < 0.001 | -0.173373 |
| SDHA | < 0.001 | -0.17342 |
| ALPK1 | < 0.001 | -0.173541 |
| TNFRSF10B | < 0.001 | -0.174084 |
| AOF2 | < 0.001 | -0.175035 |
| AC004878.3 | < 0.001 | -0.175705 |
| genomic:15-64880899-64880939 | < 0.001 | -0.176035 |
| WASH4P | < 0.001 | -0.17746 |
| SFRS18 | < 0.001 | -0.179154 |
| ARFGAP3 | < 0.001 | -0.179922 |
| GANAB | < 0.001 | -0.181451 |
| TARBP1 | < 0.001 | -0.181472 |
| MRPL37 | < 0.001 | -0.182117 |
| CMTM8 | < 0.001 | -0.182547 |
| SENP6 | < 0.001 | -0.183199 |
| QDPR | < 0.001 | -0.183302 |
| RP11-504P24.4 | < 0.001 | -0.185048 |
| PPM1G | < 0.001 | -0.188256 |
| TBC1D3C | < 0.001 | -0.188974 |
| C8B | < 0.001 | -0.190001 |
| PHB2 | < 0.001 | -0.190184 |
| TGOLN2 | < 0.001 | -0.191156 |
| AK3L1 | < 0.001 | -0.191319 |
| GM2A | < 0.001 | -0.191345 |
| LGTN | < 0.001 | -0.191511 |
| HADH | < 0.001 | -0.19266 |
| NSUN5 | < 0.001 | -0.192854 |
| VPS37C | < 0.001 | -0.193516 |
| SEC16A | < 0.001 | -0.19377 |
| CITED2 | < 0.001 | -0.194096 |
| SFRS1 | < 0.001 | -0.194962 |
| NSUN5C | < 0.001 | -0.197356 |
| AL603926.6-2 | < 0.001 | -0.198574 |
| HAUS5 | < 0.001 | -0.198773 |
| ELF1 | < 0.001 | -0.199026 |
| XRCC2 | < 0.001 | -0.200853 |
| PRPF4 | < 0.001 | -0.201045 |
| TIA1 | < 0.001 | -0.201246 |
| FKBP14 | < 0.001 | -0.201602 |
| IQCG | < 0.001 | -0.202299 |
| GPI | < 0.001 | -0.202308 |
| TRIM25 | < 0.001 | -0.20235 |
| genomic:5+69416361-69416399 | < 0.001 | -0.202891 |
| AC145146.2-1 | < 0.001 | -0.203703 |
| MAGI3 | < 0.001 | -0.203916 |
| EXOSC5 | < 0.001 | -0.204244 |
| N4BP2L2 | < 0.001 | -0.204896 |
| CDK2 | < 0.001 | -0.205157 |
| SFRS16 | < 0.001 | -0.207366 |
| RPLP2 | < 0.001 | -0.207586 |
| CCNL1 | < 0.001 | -0.207685 |
| ZFP42 | < 0.001 | -0.208984 |
| GMNN | < 0.001 | -0.210315 |
| AGPAT5 | < 0.001 | -0.210421 |
| FAM110C | < 0.001 | -0.210775 |
| MDK | < 0.001 | -0.211234 |
| ASRGL1 | < 0.001 | -0.212196 |
| ZNF202 | < 0.001 | -0.21609 |
| LARP1 | < 0.001 | -0.217343 |
| AC110285.14-1 | < 0.001 | -0.21761 |
| genomic:17-42379885-42379952 | < 0.001 | -0.21845 |
| FOXK1 | < 0.001 | -0.218641 |
| AC008268.3 | < 0.001 | -0.219726 |
| GABRE | < 0.001 | -0.219965 |
| CRABP1 | < 0.001 | -0.220304 |
| AL354696.2 | < 0.001 | -0.220545 |
| CYLC2 | < 0.001 | -0.221701 |
| BCAR3 | < 0.001 | -0.221807 |
| AC091565.10-1 | < 0.001 | -0.222143 |
| AC020663.7 | < 0.001 | -0.223726 |
| MAN1A1 | < 0.001 | -0.223938 |
| SRC | < 0.001 | -0.224959 |
| SLC8A1 | < 0.001 | -0.2282 |
| PPP3CB | < 0.001 | -0.228527 |
| C16orf70 | < 0.001 | -0.228712 |
| genomic:17+48361825-48361893 | < 0.001 | -0.229279 |
| TH1L | < 0.001 | -0.230008 |
| LUC7L | < 0.001 | -0.230893 |
| CCL25 | < 0.001 | -0.232265 |
| DNMBP | < 0.001 | -0.23308 |
| HNRPA2B1 | < 0.001 | -0.234298 |
| SULT1A4 | < 0.001 | -0.235575 |
| FNBP4 | < 0.001 | -0.235902 |
| LOXL2 | < 0.001 | -0.236463 |
| PILRB | < 0.001 | -0.237083 |
| TIGD1L | < 0.001 | -0.238024 |
| ADAM19 | < 0.001 | -0.238209 |
| LYL1 | < 0.001 | -0.238333 |
| AK3 | < 0.001 | -0.238349 |
| NACAP1 | < 0.001 | -0.241081 |
| U2AF1 | < 0.001 | -0.241733 |
| GSDMB | < 0.001 | -0.242933 |
| DERL1 | < 0.001 | -0.243076 |
| GPC3 | < 0.001 | -0.244172 |
| RIOK3 | < 0.001 | -0.245563 |
| RCBTB2 | < 0.001 | -0.247046 |
| ZNF131 | < 0.001 | -0.248379 |
| GPD1 | < 0.001 | -0.249275 |
| KIAA0907 | < 0.001 | -0.250539 |
| PAQR5 | < 0.001 | -0.251158 |
| WASF2 | < 0.001 | -0.25313 |
| MYO6 | < 0.001 | -0.25365 |
| COG3 | < 0.001 | -0.254608 |
| CDK5RAP3 | < 0.001 | -0.25538 |
| CPS1 | < 0.001 | -0.255848 |
| FZD5 | < 0.001 | -0.256734 |
| IL1R1 | < 0.001 | -0.257508 |
| PLCXD1 | < 0.001 | -0.258013 |
| SIPA1L3 | < 0.001 | -0.258052 |
| CEP135 | < 0.001 | -0.259258 |
| SOBP | < 0.001 | -0.260812 |
| SLC25A3 | < 0.001 | -0.265847 |
| SIK2 | < 0.001 | -0.266343 |
| HNRNPM | < 0.001 | -0.267847 |
| ECH1 | < 0.001 | -0.269734 |
| CRYZL1 | < 0.001 | -0.27077 |
| AC015871.7 | < 0.001 | -0.270982 |
| RP11-395L14.17 | < 0.001 | -0.272755 |
| PBX1 | < 0.001 | -0.274772 |
| VEGF | < 0.001 | -0.276281 |
| RIT2 | < 0.001 | -0.278502 |
| CLC | < 0.001 | -0.279811 |
| ERRFI1 | < 0.001 | -0.281214 |
| PCK1 | < 0.001 | -0.282794 |
| genomic:2-57217896-57217964 | < 0.001 | -0.283785 |
| genomic:14+24059672-24059706 | < 0.001 | -0.291776 |
| C1QTNF3 | < 0.001 | -0.293965 |
| TMEM212 | < 0.001 | -0.294305 |
| SNORD107 | < 0.001 | -0.295221 |
| TMEM132B | < 0.001 | -0.306597 |
| KRR1 | < 0.001 | -0.308602 |
| PCBP4 | < 0.001 | -0.30937 |
| KBTBD6 | < 0.001 | -0.311858 |
| OR8D1 | < 0.001 | -0.312165 |
| MAGT1 | < 0.001 | -0.313552 |
| ADCY2 | < 0.001 | -0.320801 |
| CDCA7 | < 0.001 | -0.321695 |
| PTK2 | < 0.001 | -0.324891 |
| KARS | < 0.001 | -0.327002 |
| HIST1H2BG | < 0.001 | -0.329912 |
| KIAA1984 | < 0.001 | -0.341268 |
| TULP2 | < 0.001 | -0.343034 |
| genomic:3-48438515-48438583 | < 0.001 | -0.345793 |
| RBM11 | < 0.001 | -0.349049 |
| genomic:1-195614916-195614984 | < 0.001 | -0.36352 |
| SCML2 | < 0.001 | -0.365665 |
| FSIP2 | < 0.001 | -0.367627 |
| DENND2A | < 0.001 | -0.371577 |
| ZNF91 | < 0.001 | -0.371866 |
| genomic:6+43504177-43504245 | < 0.001 | -0.372279 |
| IGFBP3 | < 0.001 | -0.373623 |
| SILV | < 0.001 | -0.374793 |
| ZKSCAN1 | < 0.001 | -0.383675 |
| XXbac-BPG154L12.4 | < 0.001 | -0.38803 |
| PDCL | < 0.001 | -0.389997 |
| PNN | < 0.001 | -0.390275 |
| TJP2 | < 0.001 | -0.39428 |
| NPIP | < 0.001 | -0.396858 |
| RINT1 | < 0.001 | -0.398898 |
| RHBDD1 | < 0.001 | -0.402844 |
| AHSG | < 0.001 | -0.409878 |
| ZNF14 | < 0.001 | -0.433266 |
| XXyac-R12DG2.1 | < 0.001 | -0.435728 |
| ARGLU1 | < 0.001 | -0.43859 |
| CDC42SE2 | < 0.001 | -0.442361 |
| AC233263.2-1 | < 0.001 | -0.443597 |
| genomic:7+5803135-5803170 | < 0.001 | -0.445538 |
| C9 | < 0.001 | -0.450097 |
| KIAA0556 | < 0.001 | -0.462836 |
| MTBP | < 0.001 | -0.570936 |
| AC005512.1-2 | < 0.001 | -0.582029 |
| AP000944.1 | < 0.001 | -0.699301 |
| CD14 | < 0.001 | 0.343388 |
| CSF1R | < 0.001 | 0.328228 |
| ARF5 | < 0.001 | 0.208272 |
| MXRA8 | < 0.001 | 0.200078 |
| TXNL4A | < 0.001 | 0.191668 |
| CTSD | < 0.001 | 0.174588 |
| GIPC1 | < 0.001 | 0.166936 |
| RUFY1 | < 0.001 | 0.166848 |
| SLC22A2 | < 0.001 | 0.149071 |
| AC026468.7-1 | < 0.001 | -0.134316 |
| GPR84 | < 0.001 | -0.13698 |
| KIAA0179 | < 0.001 | -0.1385 |
| CIAO1 | < 0.001 | -0.140755 |
| SEC31A | < 0.001 | -0.141258 |
| TCERG1 | < 0.001 | -0.166973 |
| ACP1 | < 0.001 | -0.188327 |
| SLC38A4 | < 0.001 | -0.201505 |
| KRT82 | < 0.001 | -0.2196 |
| TYRL | < 0.001 | -0.280961 |
| AC008869.5-1 | < 0.001 | -0.298749 |
| GSN | < 0.001 | 0.234904 |
| EEF1D | < 0.001 | 0.195605 |
| MDFI | < 0.001 | 0.183968 |
| FAM173A | < 0.001 | 0.167552 |
| C2orf29 | < 0.001 | -0.128514 |
| WSB1 | < 0.001 | -0.148578 |
| PRSS21 | < 0.001 | -0.149937 |
| ANKRD10 | < 0.001 | -0.153946 |
| CREBZF | < 0.001 | -0.160632 |
| GSTP1 | < 0.001 | 0.240778 |
| RAB31 | < 0.001 | 0.214838 |
| PLN | < 0.001 | 0.209154 |
| LYPLAL1 | < 0.001 | 0.193906 |
| QKI | < 0.001 | 0.19079 |
| genomic:17-21360637-21360705 | < 0.001 | 0.185556 |
| MMADHC | < 0.001 | 0.18359 |
| ARMET | < 0.001 | 0.18267 |
| SYNPO | < 0.001 | 0.178645 |
| IER3IP1 | < 0.001 | 0.171517 |
| OGN | < 0.001 | 0.171091 |
| CLEC11A | < 0.001 | 0.165919 |
| NGRN | < 0.001 | 0.163705 |
| PSMA3 | < 0.001 | 0.162981 |
| ZCRB1 | < 0.001 | 0.158615 |
| TBCA | < 0.001 | 0.156207 |
| C11orf73 | < 0.001 | 0.147546 |
| STX8 | < 0.001 | 0.147343 |
| TMEM50B | < 0.001 | 0.142424 |
| MYST3 | < 0.001 | 0.140318 |
| SOX18 | < 0.001 | 0.136303 |
| SERTAD3 | < 0.001 | 0.134191 |
| VPS13D | < 0.001 | -0.132977 |
| MYSM1 | < 0.001 | -0.14411 |
| UNC84A | < 0.001 | -0.145014 |
| ESM1 | < 0.001 | -0.147562 |
| SFRS18 | < 0.001 | -0.155905 |
| C6orf12 | < 0.001 | -0.160317 |
| DONSON | < 0.001 | -0.170755 |
| AP000944.1 | < 0.001 | -0.19912 |
| ATP10B | < 0.001 | -0.211137 |
| SLCO1B3 | < 0.001 | -0.345794 |
| HLA-DRB1 | 0.001 | 0.389348 |
| EFR3A | 0.001 | 0.157203 |
| MAP4K2 | 0.001 | 0.155359 |
| DPY19L4 | 0.001 | 0.148674 |
| ACTR2 | 0.001 | -0.142267 |
| RBM6 | 0.001 | -0.152659 |
| TPX2 | 0.001 | -0.20971 |
| ACTA2 | 0.001 | 0.328825 |
| APOD | 0.001 | 0.198873 |
| HOXB7 | 0.001 | 0.16739 |
| ASF1A | 0.001 | 0.154525 |
| PYCRL | 0.001 | 0.153974 |
| ACSBG1 | 0.001 | 0.149273 |
| RAPGEF4 | 0.001 | 0.139441 |
| PTPRA | 0.001 | 0.13717 |
| TUSC2 | 0.001 | 0.135669 |
| TMEM219 | 0.001 | 0.134658 |
| RHOD | 0.001 | -0.123482 |
| RP11-545I5.3 | 0.001 | -0.134071 |
| ARPC4 | 0.001 | -0.135805 |
| AC126564.7 | 0.001 | -0.142728 |
| AHSA2 | 0.001 | -0.144559 |
| CCDC122 | 0.001 | -0.15518 |
| RNF213 | 0.001 | -0.155816 |
| FOS | 0.001 | -0.338612 |
| CETN3 | 0.001 | 0.167827 |
| SERTAD1 | 0.001 | 0.133475 |
| MCM7 | 0.001 | -0.18169 |
| INSIG1 | 0.001 | -0.295002 |
| ITGB2 | 0.002 | 0.248018 |
| EPHA3 | 0.002 | 0.241585 |
| NME1-NME2 | 0.002 | 0.214904 |
| NAB1 | 0.002 | 0.1959 |
| SLC7A7 | 0.002 | 0.189545 |
| PPP1R2 | 0.002 | 0.180946 |
| FILIP1L | 0.002 | 0.160566 |
| SLC27A1 | 0.002 | 0.149278 |
| C21orf91 | 0.002 | 0.147539 |
| ATP6V1D | 0.002 | 0.145226 |
| RBX1 | 0.002 | 0.143138 |
| EHD1 | 0.002 | 0.126762 |
| ATG9B | 0.002 | -0.119224 |
| KLRC3 | 0.002 | -0.136231 |
| NUDC | 0.002 | -0.145921 |
| TBC1D4 | 0.002 | -0.168002 |
| AP1GBP1 | 0.002 | -0.185862 |
| ADA | 0.002 | -0.259567 |
| YIPF5 | 0.002 | 0.176941 |
| TRIM13 | 0.002 | 0.173605 |
| HSF1 | 0.002 | 0.17062 |
| IFI27L2 | 0.002 | 0.166931 |
| NUDT3 | 0.002 | 0.157117 |
| ANXA11 | 0.002 | 0.153948 |
| HS3ST2 | 0.002 | 0.147074 |
| PEF1 | 0.002 | 0.12097 |
| TUBE1 | 0.002 | -0.126073 |
| COL12A1 | 0.002 | -0.144809 |
| CEACAM8 | 0.002 | -0.1536 |
| ATXN3 | 0.002 | -0.159005 |
| SFRS2 | 0.002 | -0.171244 |
| HBZ | 0.002 | -0.315253 |
| VSIG4 | 0.002 | 0.240838 |
| DDAH2 | 0.002 | 0.218349 |
| TM9SF3 | 0.002 | 0.210741 |
| NCF2 | 0.002 | 0.20911 |
| CHIT1 | 0.002 | 0.201889 |
| MS4A6A | 0.002 | 0.187403 |
| GRN | 0.002 | 0.177911 |
| USP14 | 0.002 | 0.16896 |
| MAPRE1 | 0.002 | 0.161142 |
| ICMT | 0.002 | 0.146348 |
| NDUFB8 | 0.002 | 0.135422 |
| POLB | 0.002 | 0.134877 |
| ZBBX | 0.002 | 0.123796 |
| genomic:19+29180695-29180763 | 0.002 | -0.113298 |
| ALS2 | 0.002 | -0.12235 |
| MYO1E | 0.002 | -0.136151 |
| ANAPC7 | 0.002 | -0.138672 |
| TFDP1 | 0.002 | -0.141073 |
| APBB2 | 0.002 | -0.14688 |
| U3 | 0.002 | -0.150412 |
| ITGAM | 0.002 | 0.249907 |
| SYS1 | 0.002 | 0.233451 |
| BACH2 | 0.002 | 0.193574 |
| C2orf12 | 0.002 | 0.186038 |
| TCEAL4 | 0.002 | 0.160741 |
| CCDC92 | 0.002 | 0.151228 |
| ZMAT2 | 0.002 | 0.150724 |
| EXOC6 | 0.002 | 0.13324 |
| ARRB2 | 0.002 | 0.123332 |
| CPT2 | 0.002 | -0.120383 |
| FZD7 | 0.002 | -0.13481 |
| DDX56 | 0.002 | -0.138967 |
| TBRG1 | 0.002 | -0.140407 |
| PRDX6 | 0.002 | -0.155143 |
| RGS5 | 0.002 | -0.193762 |
| TAX1BP3 | 0.003 | 0.225864 |
| CD47 | 0.003 | 0.167877 |
| JAK3 | 0.003 | -0.123217 |
| CBS | 0.003 | -0.180311 |
| CPNE3 | 0.003 | 0.180256 |
| PAN2 | 0.003 | -0.130857 |
| GMDS | 0.003 | -0.147961 |
| MAP4K4 | 0.003 | -0.155064 |
| SLC38A2 | 0.003 | -0.219405 |
| C13orf15 | 0.003 | 0.221349 |
| U2AF1 | 0.003 | -0.128828 |
| PARK7 | 0.003 | 0.161697 |
| SRPRB | 0.003 | 0.148255 |
| AC068288.1 | 0.003 | -0.114384 |
| RBM33 | 0.003 | -0.135831 |
| UBE2D1 | 0.004 | 0.14177 |
| LRRCC1 | 0.004 | 0.215754 |
| S100A11P | 0.004 | 0.191126 |
| CDK2AP1 | 0.004 | 0.186404 |
| IQGAP1 | 0.004 | 0.168413 |
| SLAMF8 | 0.004 | 0.163548 |
| ZNF268 | 0.004 | 0.14492 |
| UBE2E2 | 0.004 | 0.139553 |
| CD59 | 0.004 | 0.130326 |
| C18orf21 | 0.004 | 0.123769 |
| SPG21 | 0.004 | 0.122743 |
| CDS2 | 0.004 | 0.120319 |
| AC124287.10 | 0.004 | -0.129204 |
| AIPL1 | 0.004 | -0.137954 |
| RP3-452M16.1 | 0.004 | -0.160251 |
| DPT | 0.004 | 0.166451 |
| ASPN | 0.004 | 0.395696 |
| DEFA6 | 0.004 | 0.39004 |
| MS4A6E | 0.004 | 0.260201 |
| NBPF1 | 0.004 | 0.179561 |
| SLC1A3 | 0.004 | 0.153503 |
| MARK2 | 0.004 | 0.126078 |
| CALHM2 | 0.004 | 0.122475 |
| SLC43A3 | 0.004 | -0.117218 |
| NEDD8 | 0.004 | -0.165188 |
| COL4A5 | 0.004 | -0.176086 |
| FAP | 0.004 | 0.190836 |
| NRIP1 | 0.004 | 0.160966 |
| CDK4 | 0.004 | 0.14369 |
| HERC4 | 0.004 | -0.126748 |
| MMD | 0.005 | 0.159041 |
| PSMB2 | 0.005 | -0.135245 |
| RBBP7 | 0.005 | -0.217858 |
| CFD | 0.005 | 0.233842 |
| CHMP1A | 0.005 | 0.132395 |
| OGFOD2 | 0.005 | -0.11802 |
| genomic:12-112142226-112142294 | 0.005 | -0.122342 |
| H2AFY | 0.005 | -0.126486 |
| SLU7 | 0.005 | -0.144556 |
| genomic:GL000205.1+45142-45210 | 0.005 | -0.186212 |
| PRAME | 0.005 | -0.230958 |
| TALDO1 | 0.006 | 0.158597 |
| FAM172A | 0.006 | 0.141075 |
| SDF2 | 0.006 | 0.126125 |
| RHOXF2 | 0.006 | -0.237364 |
| PLEKHB1 | 0.006 | 0.151217 |
| WBSCR1 | 0.006 | -0.133997 |
| BAT2D1 | 0.006 | -0.151343 |
| CDC42SE2 | 0.007 | 0.178731 |
| MMP25 | 0.007 | 0.162246 |
| DNTTIP2 | 0.007 | 0.12561 |
| SFRS2IP | 0.007 | -0.133279 |
| SPIRE1 | 0.007 | -0.153343 |
| CD46 | 0.007 | -0.244792 |
| PITRM1 | 0.007 | -0.122006 |
| NOS1 | 0.007 | -0.137415 |
| MED13 | 0.007 | -0.146236 |
| RANBP1 | 0.007 | -0.194992 |
| SLC39A4 | 0.007 | 0.18372 |
| TNKS1BP1 | 0.007 | 0.119025 |
| EVI5 | 0.007 | -0.127791 |
| POP4 | 0.007 | 0.113743 |
| AP000640.5-1 | 0.007 | 0.264503 |
| IFI30 | 0.007 | 0.229473 |
| CTNND1 | 0.007 | 0.150006 |
| C21orf7 | 0.007 | 0.147034 |
| DLG1 | 0.007 | -0.133555 |
| AP4B1 | 0.007 | -0.149776 |
| SORD | 0.007 | -0.15901 |
| RP11-436K8.1 | 0.007 | -0.177853 |
| COPS5 | 0.008 | 0.164894 |
| ZNF588 | 0.008 | -0.191185 |
| TMC5 | 0.008 | -0.226496 |
| TSC22D3 | 0.008 | 0.219499 |
| SF3B5 | 0.008 | 0.158386 |
| COMP | 0.008 | 0.149513 |
| CD300A | 0.008 | 0.148836 |
| SEC16B | 0.008 | -0.161135 |
| STOML2 | 0.009 | -0.162643 |
| OXA1L | 0.009 | -0.173028 |
| SIVA1 | 0.009 | 0.15502 |
| COX17 | 0.009 | 0.133679 |
| DNAJB11 | 0.010 | 0.134097 |
| COPS4 | 0.010 | 0.128481 |
| CNTNAP3 | 0.010 | -0.118917 |
| SFRS14 | 0.010 | -0.119222 |
| TAOK1 | 0.010 | -0.123089 |
| RP11-397O8.4 | 0.010 | 0.223815 |
| PPP3CB | 0.010 | 0.137012 |
| PRKRA | 0.010 | -0.140415 |
| HTATIP2 | 0.010 | -0.167465 |
| NA | 0.011 | -0.123948 |
| genomic:10-1099812-1099880 | 0.011 | -0.126591 |
| ZNF623 | 0.011 | -0.146901 |
| ACADM | 0.011 | -0.176457 |
| ACOX2 | 0.011 | -0.204663 |
| POLA2 | 0.011 | -0.128493 |
| CNOT7 | 0.011 | 0.208271 |
| RELL1 | 0.011 | 0.157614 |
| GAA | 0.011 | 0.129986 |
| DDX17 | 0.011 | -0.139425 |
| genomic:12+32536391-32536459 | 0.012 | 0.141704 |
| GABARAPL2 | 0.012 | 0.130164 |
| MEF2C | 0.012 | 0.170683 |
| ORMDL2 | 0.012 | 0.112995 |
| KBTBD8 | 0.012 | 0.109083 |
| C6orf89 | 0.012 | -0.112763 |
| S100A4 | 0.012 | 0.270248 |
| ATP5H | 0.012 | 0.148272 |
| PLCD3 | 0.012 | 0.107668 |
| THPO | 0.012 | -0.136544 |
| TOR1AIP2 | 0.012 | -0.156076 |
| SERPINA7 | 0.012 | -0.213522 |
| PTDSS1 | 0.012 | 0.154841 |
| GABARAP | 0.012 | 0.131055 |
| PRPF38A | 0.012 | -0.132492 |
| SLC38A3 | 0.012 | -0.185133 |
| CAPZA2 | 0.013 | 0.196522 |
| TTC1 | 0.013 | 0.124808 |
| RP5-854E16.1 | 0.013 | -0.118095 |
| UBE2Q1 | 0.013 | -0.292936 |
| SKIV2L2 | 0.014 | 0.138501 |
| CYP3A5 | 0.014 | -0.112554 |
| EXOSC1 | 0.014 | 0.128965 |
| RNF13 | 0.014 | 0.121707 |
| SPTAN1 | 0.014 | -0.114985 |
| SF3B3 | 0.014 | -0.127469 |
| AZGP1 | 0.014 | -0.308959 |
| genomic:1+64050224-64050292 | 0.014 | 0.1579 |
| TMED9 | 0.014 | 0.135758 |
| SPG21 | 0.014 | -0.126299 |
| BLMH | 0.014 | -0.137372 |
| IER5 | 0.014 | -0.163754 |
| PFKM | 0.015 | -0.124674 |
| RNF181 | 0.015 | 0.12795 |
| RELN | 0.015 | -0.140821 |
| RHOC | 0.016 | 0.166175 |
| RRM2B | 0.016 | 0.142468 |
| MRPS28 | 0.016 | 0.115343 |
| GPR172A | 0.016 | 0.168672 |
| STC2 | 0.016 | -0.150174 |
| SLC17A7 | 0.016 | -0.183985 |
| IFI6 | 0.017 | 0.399226 |
| NDUFB7 | 0.017 | 0.116195 |
| SLC30A1 | 0.017 | -0.125767 |
| PI4KA | 0.017 | -0.169717 |
| ZFR | 0.017 | 0.116855 |
| SVIL | 0.017 | -0.124322 |
| LUC7L2 | 0.017 | -0.139062 |
| ZNF121 | 0.017 | -0.359435 |
| GNAI2 | 0.017 | 0.163855 |
| FAM49B | 0.018 | 0.16355 |
| GSTM3 | 0.018 | 0.270821 |
| HMBOX1 | 0.018 | -0.113974 |
| ATF4 | 0.019 | -0.140876 |
| VCAM1 | 0.019 | 0.190154 |
| STK38 | 0.019 | -0.167862 |
| CSAG3 | 0.019 | -0.431002 |
| AMD1 | 0.019 | 0.12544 |
| ACO1 | 0.019 | -0.112245 |
| SDR39U1 | 0.019 | -0.148529 |
| SERPIND1 | 0.019 | -0.25324 |
| CTSS | 0.019 | 0.239414 |
| RBM18 | 0.019 | 0.11904 |
| AC091849.2-1 | 0.019 | -0.129848 |
| ZNF274 | 0.020 | 0.149083 |
| FIS1 | 0.021 | 0.180842 |
| IQCH | 0.021 | 0.151677 |
| ZNHIT2 | 0.021 | 0.134755 |
| FKBP1B | 0.021 | -0.21594 |
| SSR3 | 0.021 | 0.118645 |
| SOD2 | 0.021 | -0.124047 |
| FCGR2B | 0.021 | 0.206397 |
| KIAA0040 | 0.021 | 0.136293 |
| MRPS22 | 0.021 | 0.131075 |
| YTHDC1 | 0.021 | -0.116253 |
| CRNN | 0.022 | 0.135076 |
| RP4-581F12.2 | 0.022 | 0.124732 |
| DAPK3 | 0.022 | 0.119715 |
| FNTA | 0.022 | 0.182173 |
| SLIT3 | 0.022 | -0.143049 |
| CCL3 | 0.023 | 0.242637 |
| ZNF611 | 0.023 | -0.1079 |
| NGLY1 | 0.023 | -0.143987 |
| SPAG5 | 0.023 | 0.180646 |
| PSIP1 | 0.023 | 0.169831 |
| FVT1 | 0.023 | 0.153943 |
| LLGL2 | 0.023 | 0.127875 |
| IL2RG | 0.024 | 0.15185 |
| TTTY7 | 0.024 | 0.139818 |
| HTRA1 | 0.025 | 0.278675 |
| EML4 | 0.025 | -0.178585 |
| ROCK1 | 0.025 | -0.133597 |
| YWHAZ | 0.026 | 0.13388 |
| UBE2Z | 0.026 | 0.118205 |
| CD8BP | 0.026 | -0.305308 |
| CREBL2 | 0.026 | 0.173964 |
| MGAT2 | 0.027 | 0.110992 |
| ZNF614 | 0.028 | -0.128392 |
| EXT1 | 0.028 | -0.200233 |
| C4orf18 | 0.028 | 0.223548 |
| S100A13 | 0.028 | 0.123432 |
| AURKA | 0.028 | -0.150518 |
| FOLR3 | 0.029 | -0.113739 |
| LY86 | 0.029 | 0.138874 |
| GTF2A2 | 0.029 | 0.14975 |
| ROCK2 | 0.029 | -0.189896 |
| CNBP | 0.029 | 0.109054 |
| RBP7 | 0.030 | 0.111739 |
| CBWD2 | 0.030 | -0.143738 |
| IL7R | 0.032 | 0.138124 |
| NCAPG2 | 0.032 | -0.144959 |
| ABHD12 | 0.032 | 0.138442 |
| SIRPA | 0.032 | 0.121444 |
| GATAD1 | 0.032 | -0.111227 |
| MOXD1 | 0.033 | 0.190197 |
| RP11-480I12.9 | 0.033 | 0.140645 |
| CDC16 | 0.033 | -0.128238 |
| DAP3 | 0.033 | -0.141483 |
| SYNPO2 | 0.033 | 0.191143 |
| AREGB | 0.033 | 0.178348 |
| KNTC1 | 0.033 | -0.116171 |
| VPS13A | 0.033 | -0.137167 |
| TGFB3 | 0.034 | 0.155567 |
| TMX1 | 0.034 | 0.125519 |
| SCGB1D2 | 0.034 | 0.174542 |
| RP11-345P4.4 | 0.034 | -0.122775 |
| ZNF37A | 0.034 | -0.162986 |
| KRT15 | 0.034 | -0.117362 |
| NUP155 | 0.034 | -0.132323 |
| MDH2 | 0.035 | -0.184061 |
| ANAPC11 | 0.035 | 0.149745 |
| CD83 | 0.035 | 0.124599 |
| GLT8D2 | 0.035 | 0.110106 |
| CARD6 | 0.035 | 0.142974 |
| NUS1 | 0.036 | 0.130699 |
| NAGA | 0.036 | 0.119116 |
| TNFRSF18 | 0.036 | 0.143307 |
| RP11-93B14.6 | 0.036 | -0.172717 |
| UBL5 | 0.037 | 0.127561 |
| NKTR | 0.038 | -0.141155 |
| NA | 0.038 | 0.156122 |
| XRCC1 | 0.038 | 0.148644 |
| CBX5 | 0.038 | -0.114554 |
| ASS1 | 0.039 | -0.289711 |
| TCEAL8 | 0.039 | 0.163485 |
| DLK1 | 0.039 | -0.133012 |
| genomic:10+70460150-70460218 | 0.040 | 0.158913 |
| AP3S2 | 0.041 | 0.153683 |
| HAMP | 0.041 | -0.332968 |
| ASPH | 0.042 | 0.139692 |
| AC009086.6-1 | 0.042 | 0.162688 |
| IARS | 0.042 | -0.15188 |
| PRKAB2 | 0.043 | -0.102741 |
| PAK1IP1 | 0.043 | 0.151653 |
| LILRB2 | 0.043 | 0.209717 |
| genomic:1+182582985-182583053 | 0.044 | 0.150642 |
| ACYP1 | 0.044 | -0.161306 |
| ICOSLG | 0.044 | 0.104937 |
| CHRFAM7A | 0.045 | -0.140054 |
| RAD21L1 | 0.045 | -0.180417 |
| SDCBP | 0.045 | 0.182232 |
| NBPF4 | 0.045 | 0.143871 |
| WDR77 | 0.046 | -0.132862 |
| CCDC6 | 0.046 | 0.156356 |
| NUP88 | 0.046 | 0.147718 |
| MAP3K7IP2 | 0.047 | 0.129909 |
| YPEL5 | 0.047 | 0.141696 |
| SPTBN1 | 0.048 | 0.14493 |
| COMMD3 | 0.048 | 0.131095 |
| CDCA3 | 0.048 | -0.141512 |
| GTF2H3 | 0.048 | -0.111046 |
| PRKAG2 | 0.050 | -0.107397 |

^a^ P values adjusted for multiple testing with Benjamini-Hochberg.
