# Supplementary material for: Exploring Gene Expression Signatures for Predicting Disease Free Survival after Resection of Colorectal Cancer Liver Metastases
Source: PLoS One. 2012 Nov 21;7(11):e49442. doi: 10.1371/journal.pone.0049442 (PMC3504021; doi:10.1371/journal.pone.0049442)
Supplement: Table S5 — Signature performances for predicting DFS as a dichotomous outcome.a (DOC) [file pone.0049442.s006.doc]

**Table S5: Signature performances for predicting DFS as a dichotomous outcome^a^**

|  | **Training**  **All^b^** | **Validation**  **All^c^** | **Validation**  **Strict^d^** | **Validation**  **UMCU^e^** | **Validation**  **PB^f^** | **Validation**  **PB treated^g^** |
| --- | --- | --- | --- | --- | --- | --- |
| **Accuracy** | 87% | 57% | 61% | 41% | 63% | 58% |
| **Sensitivity** | 85% | 58% | 67% | 38% | 63% | 64% |
| **Specificity** | 89% | 55% | 55% | 44% | 64% | 40% |
| **PPV** | 93% | 61% | 64% | 38% | 71% | 75% |
| **NPV** | 77% | 52% | 58% | 44% | 54% | 29% |
| **AUC** | 0.87 | 0.57 | 0.61 | 0.41 | 0.63 | 0.52 |
| **OR^h^**  **(P value/corrected P value)** | 43.02  (<0.001/<0.001) | 1.69  (0.55/0.77) | 2.39  (0.22/0.50) | 0.5  (0.64/0.77) | 2.8  (0.25/0.50) | 1.2  (1/1) |

DFS, disease free survival; UMCU, UMC Utrecht; PB, Paul Brousse; PPV, positive predictive value; NPV, negative predictive value; AUC, area under the curve; OR, odds ratio

**^a^** High risk (DFS ≤ 1 year) vs low risk (DFS > 1 year).

**^b^** Signature trained on all samples in training set and validated on all samples in training set.

**^c^** Signature trained on all samples in training set and validated on all samples in validation set.

**^d^** Signature trained on samples in training set, validated on samples in validation set. For the training step high risk was defined as DFS ≤ 6 months and low risk as DFS > 24 months.

**^e^** Signature trained on UMC Utrecht samples in training set and validated on UMC Utrecht samples in validation set.

**^f^** Signature trained on Paul Brousse samples in training set and validated on Paul Brousse samples in validation set.

**^g^** Signature trained on Paul Brousse samples treated with neoadjuvant chemotherapy in training set and validated on Paul Brousse samples treated with neoadjuvant chemotherapy in validation set.

**^h^** Odds ratio for DFS ≤ 1 year (High Risk prediction vs Low Risk prediction). P values are based on Fisher’s exact test.
